# Dynamics of BMP signaling and stable gene expression in the early *Drosophila* embryo

**DOI:** 10.1242/bio.061646

**Published:** 2024-08-29

**Authors:** Hadel Al Asafen, Aydin Beseli, Hung-Yuan Chen, Sharva Hiremath, Cranos M. Williams, Gregory T. Reeves

**Affiliations:** ^1^ Department of Chemical and Biomolecular Engineering, North Carolina State University, Raleigh, NC 27695, USA; ^2^ Department of Chemical Engineering, Texas A&M University, College Station, TX 77843, USA; ^3^ Department of Electrical and Computer Engineering, North Carolina State University, Raleigh, NC 27695, USA; ^4^ North Carolina Plant Sciences Initiative, North Carolina State University, Raleigh, NC 27695, USA; ^5^ Interdisciplinary Graduate Program in Genetics, Texas A&M University, College Station, TX 77843, USA

**Keywords:** BMP signaling, *Drosophila* embryo, Medea, Live imaging, Morphogen gradient, Memory

## Abstract

In developing tissues, morphogen gradients are thought to initialize gene expression patterns. However, the relationship between the dynamics of morphogen-encoded signals and gene expression decisions is largely unknown. Here we examine the dynamics of the Bone Morphogenetic Protein (BMP) pathway in *Drosophila* blastoderm-stage embryos. In this tissue, the BMP pathway is highly dynamic: it begins as a broad and weak signal on the dorsal half of the embryo, then 20-30 min later refines into a narrow, intense peak centered on the dorsal midline. This dynamical progression of the BMP signal raises questions of how it stably activates target genes. Therefore, we performed live imaging of the BMP signal and found that dorsal-lateral cells experience only a short transient in BMP signaling, after which the signal is lost completely. Moreover, we measured the transcriptional response of the BMP target gene *pannier* in live embryos and found it to remain activated in dorsal-lateral cells, even after the BMP signal is lost. Our findings may suggest that the BMP pathway activates a memory, or ‘ratchet’ mechanism that may sustain gene expression.

## Teaser

**Summary:** The BMP signaling pathway is highly dynamic in the early *Drosophila* embryo. Live cell imaging quantifies these dynamics and points to a memory mechanism to regulate gene expression.

## INTRODUCTION

In developing tissues, gene expression decisions are initiated by morphogen gradients, which are often assumed to deliver a continuous input of a steady signal (Gurdon and Bourillot, 2001; Wharton et al., 2004; Jaeger et al., 2004; Reeves et al., 2005; Ashe and Briscoe, 2006; Dresch et al., 2013). However, the dynamics of morphogen gradients may also play a role in gene expression (Delotto et al., 2007; Gregor et al., 2007; Nahmad and Stathopoulos, 2009; Ribes et al., 2010; Reeves et al., 2012). In the past two decades, studies using green fluorescent protein (GFP)-tagged morphogens – including early *Drosophila* morphogens Bicoid and Dorsal; Dpp in the wing imaginal disc; and the Hedgehog, Wnt, and TGF-β families in vertebrates – have revealed that the establishment of morphogen gradients is a highly dynamic and complex process (Delotto et al., 2007; Entchev et al., 2000; Gregor et al., 2007; Holzer et al., 2012; Luz et al., 2014; Reeves et al., 2012; Ribes et al., 2010; Teleman and Cohen, 2000; Wallkamm et al., 2014; Wartlick et al., 2011; Williams et al., 2004; Zhou et al., 2012). Live imaging studies – of both morphogen input and the resulting gene expression output – have challenged the established view that tissue patterning relies on a constant, steady-state level of signaling to regulate gene expression (Aldaz et al., 2010; Ashe and Briscoe, 2006; Dresch et al., 2013; Gurdon and Bourillot, 2001; Gurdon et al., 1995; Jaeger et al., 2004; Mavrakis et al., 2010; Oates et al., 2009; Pantazis and Supatto, 2014; Reeves et al., 2005; Wharton et al., 2004).

**Fig. 1.**
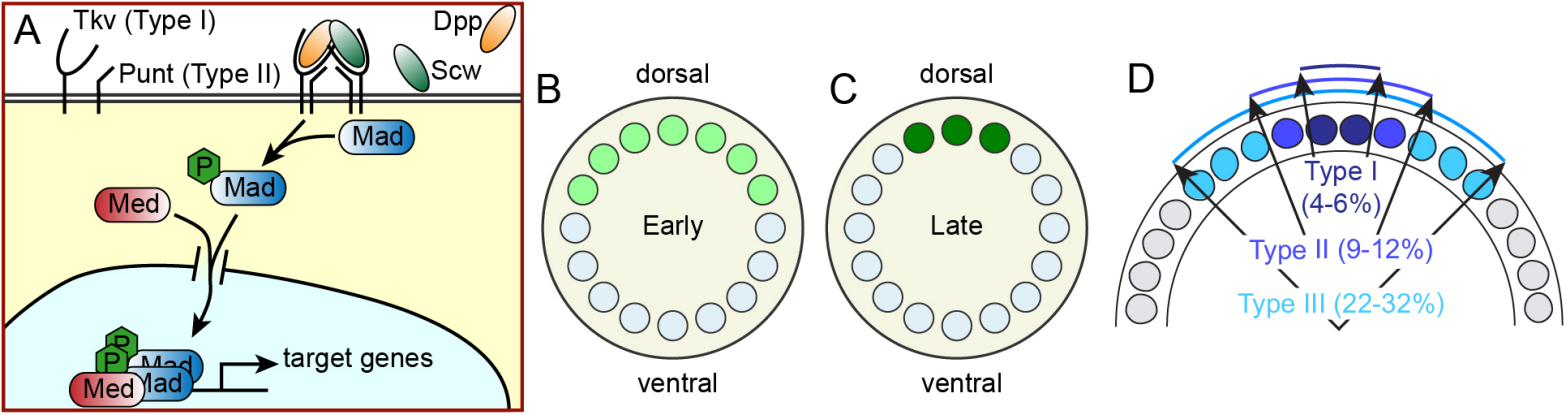
**Schematic of the BMP network.** (A) BMP ligands Dpp and Scw bind to their cognate Type I receptors, which dimerize with Type II receptors. The active receptor signaling complex causes the phosphorylation of Mad, which partners with Med to enter the nucleus. (B) The early stage of BMP signaling is broad and weak. (C) Roughly 20 min later, BMP signaling has intensified into a narrow domain. (D) BMP target genes, denoted Types I-III, are expressed in three nested domains. The ventral boundaries of these domains are noted on the figure.

The bone morphogenetic protein (BMP) signaling pathway in the blastoderm stage *Drosophila* embryo is a classic example of a highly dynamic morphogen-mediated system. BMPs are a group of signaling molecules that were initially discovered for their ability to induce bone formation. BMPs are now known to play crucial roles in many tissue-patterning processes, including establishing the dorsal-most tissues during *Drosophila* embryogenesis. Roughly 2 h after fertilization, when the nuclei have completed 13 nuclear division cycles, ∼6000 nuclei are present at the embryo's cortex. During the 14th interphase [nuclear cycle (nc) 14], BMP signaling acts through two of the *Drosophila* BMP ligands, Dpp (expressed on the dorsal 50% of the embryo) and Screw (Scw; uniformly expressed) (Padgett et al., 1987; Arora et al., 1994). The ligand-bound Type I receptors, Thickveins and Saxophone (Brummel et al., 1994), together with Punt (a Type II receptor) (Letsou et al., 1995), phosphorylate Mothers against Dpp (Mad; homolog of vertebrate Smad1), which enters the nucleus with Co-Smad Medea (Med; homolog of Smad4) to regulate gene expression (Fig. 1A) (Raftery et al., 1995; Raftery and Sutherland, 2003).

The spatial pattern of BMP signaling is highly dynamic, and has been assayed by immunostaining of the phosphorylated form of Mad (pMad) in fixed embryos (Tanimoto et al., 2000; Ross et al., 2001; Rushlow et al., 2001; Dorfman and Shilo, 2001; Raftery and Sutherland, 2003; Wang and Ferguson, 2005; O'Connor et al., 2006; Gavin-Smyth et al., 2013). In early nc 14, the initial pattern of BMP signaling is broad and weak, covering the dorsal 30-40% of the embryo (Fig. 1B). Just 20-30 min later, the BMP signal intensifies and sharpens to a narrow domain in the dorsal-most 5-6% of the embryo (Fig. 1C) (Raftery and Sutherland, 2003; O'Connor et al., 2006; Gavin-Smyth et al., 2013). The broad, low intensity signal likely directs the dorsal half of the embryo to become dorsal epidermis, and the cells in the domain of late, high intensity signal eventually develop into the amnioserosa (Bier and De Robertis, 2015).

How cells interpret this dynamic BMP signal to form stable gene-expression patterns, and whether the precise progression of BMP dynamics is necessary for proper development, is unknown. Three nested domains of BMP target genes (Types I-III) have been observed (Fig. 1D) (Ashe et al., 2000; Xu et al., 2005; Lin et al., 2006; Liang et al., 2012). However, at no single stage do quantitative data suggest the BMP profile has the ability to simultaneously specify all three domains: the early, broad domain is too weak to activate Type I genes, while the refined state is too narrow to maintain Type III genes (Gavin-Smyth et al., 2013). Moreover, detailed studies of the regulatory units of BMP target genes have raised additional questions, as no clear affinity-based enhancer structure has been found (Xu et al., 2005; Lin et al., 2006; Liang et al., 2012). In particular, qualitative studies have noted that the Type III domain is not clearly correlated with the pMad domain (Xu et al., 2005).

The best-studied Type III gene, *pannier* (*pnr*), has a complex expression pattern restricted to the 25% dorsal-most cells that lie between 40% and 80% AP (0% AP being the anterior pole) (Liang et al., 2012). *pnr* expression is composed of two distinctly regulated domains: a contiguous ‘dorsal patch’ of cells (Liang et al., 2012), and six AP-modulated stripes overlaid on the patch. Regulation of the patch region is partially separable from the stripes, as an enhancer for *pnr* was isolated from the first intron of *pnr*. This enhancer, denoted the *pnr P3* enhancer, drives the expression of the dorsal patch and two of the six stripes (Liang et al., 2012). The patch requires BMP signaling, as it is lost in *dpp* mutants. The stripes become dramatically weaker in these mutants as well (Liang et al., 2012).

In this paper, we measured the dynamics of both BMP signaling and *pnr* expression in live embryos. We first showed in fixed embryos that nuclear localization of Med-GFP is an appropriate assay for BMP signaling, as it correlates strongly with pMad staining. Next, we imaged the dynamics of Med-GFP in live embryos and showed that cells in the tails of the Type III domain [between 9% and 25% dorsal-ventral (DV) coordinate] experience only a transient of BMP signaling in the first half of nc 14. Finally, we showed in both live and fixed embryos that the cells in this same domain continue to express *pnr* throughout nc 14. We argue that *pnr* requires BMP for activation, but not maintenance. We hypothesize this may constitute a memory, or ‘ratchet’, module, similar to that found in Hedgehog signaling in the wing disc or Dorsal activity in DV patterning of the early embryo (Nahmad and Stathopoulos, 2009; Irizarry et al., 2020).

## RESULTS AND DISCUSSION

### Nuclear Med-GFP mirrors pMad

**Fig. 2.**
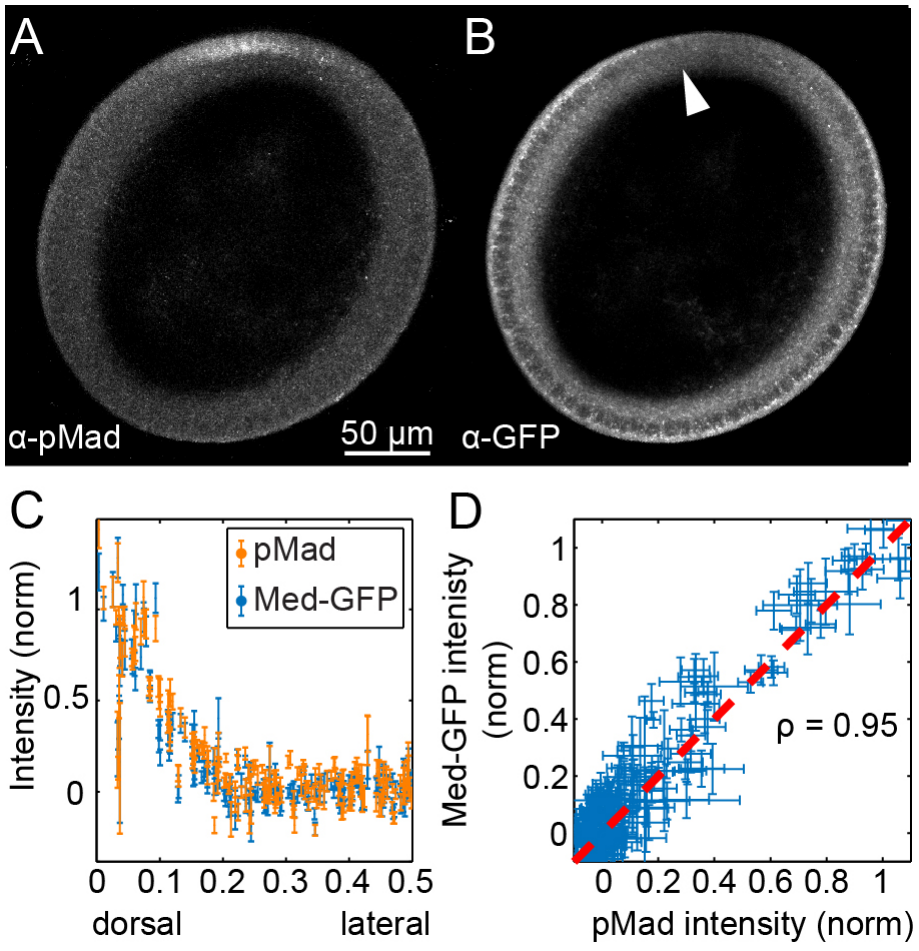
**Correlation between pMad and nuclear Med-GFP**. (A,B) Cross section of nc 14 embryo expressing Med-GFP and immunostained against pMad (A) and GFP (B); scale bar: 50µm. (C) Quantification of normalized nuclear intensities for 15 z-slices of embryo shown in A and B. The nuclear intensities of pMad and Med-GFP are highly correlated. (D) Plot of the Med-GFP normalized nuclear intensity versus the pMad normalized nuclear intensity. Error bars indicate s.e.m. All analyzed embryos (*n*=8) can be found in Fig. S2.

In cells with activated BMP signaling, Mad is phosphorylated and both pMad and Med enter the nucleus (Fig. 1A). To determine whether nuclear localization of Med-GFP is an appropriate assay for BMP signaling in live embryos, we first measured pMad and nuclear Med-GFP in fixed, nc 14 embryos (see Materials and Methods). Nuclear localized Med-GFP was higher on the dorsal side, in the same nuclei as the pMad staining (Fig. 2A,B). While nuclear Med-GFP was not necessarily higher than cytoplasmic Med-GFP in dorsal nuclei, in the rest of the embryo Med-GFP was excluded from the nuclei. Late Stage 5/early Stage 6 embryos do in fact exhibit higher levels of nuclear Med-GFP than the surrounding cytoplasm (Sutherland et al., 2003) (Fig. S1).

To visualize the overlap between the pMad and Med-GFP stainings, we plotted their normalized nuclear intensities on the same axes (Fig. 2C; Fig. S2). We normalized their nuclear intensities by fitting them to Gaussian-shaped curves (Liberman et al., 2009; Trisnadi et al., 2013) (see ‘Empirical Fitting’ section of Supplemental Materials and Methods). Visually, these plots showed a strong overlap between pMad and the nuclear intensity of Med-GFP. The high correlation (ρ=0.9±0.05 s.d.; *n*=8) becomes more clear when the normalized intensity of Med-GFP is plotted against that of pMad for each nucleus (Fig. 2D, Fig. S2). Therefore, these data in fixed embryos support using nuclear localization of Med-GFP in live embryos as a readout of BMP signaling.

### Measurements of nuclear Med-GFP dynamics

**Fig. 3.**
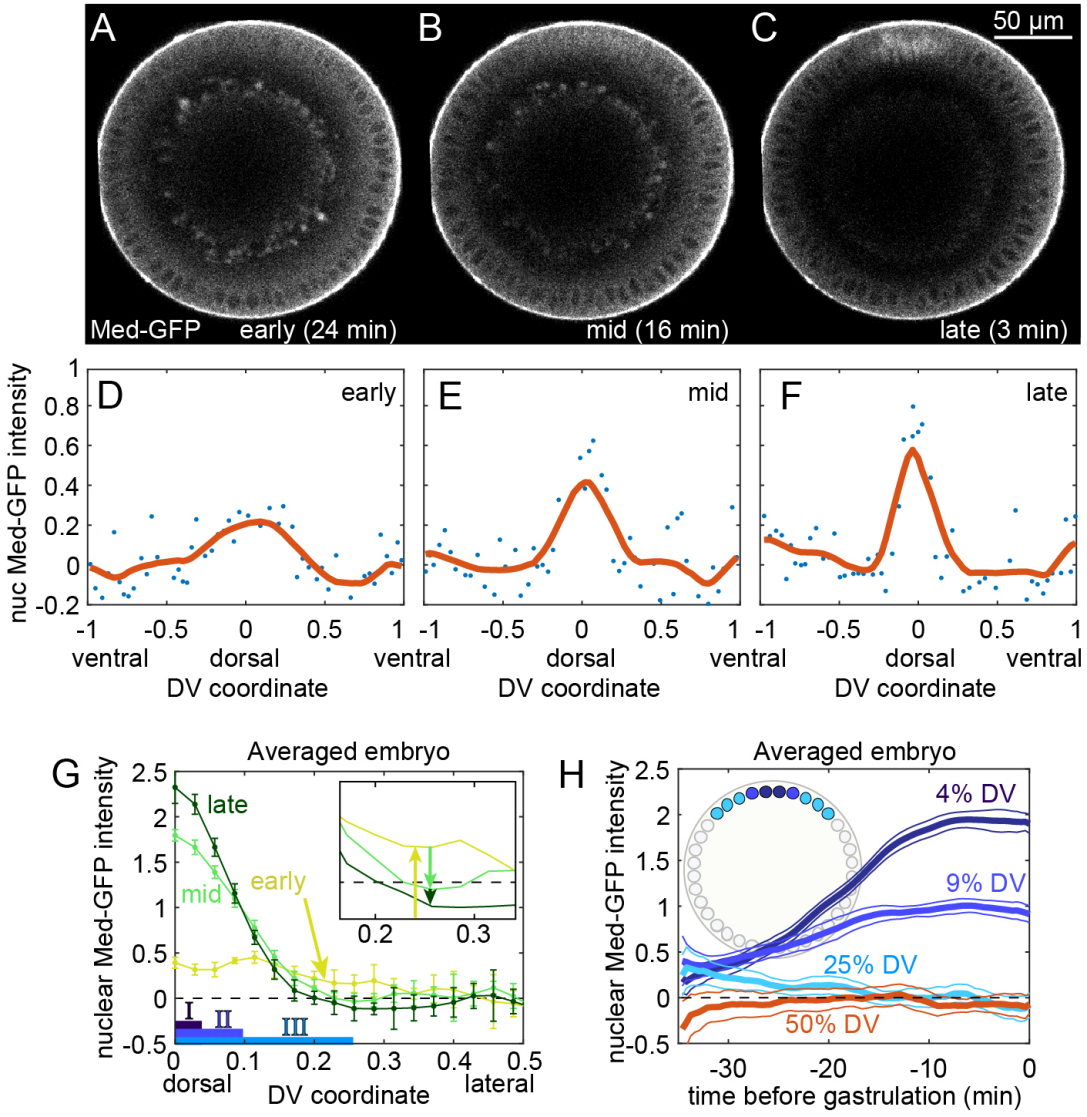
**Dynamics of Med-GFP**. (A-C) Snapshots of live embryo expressing Med-GFP. Time stamps for early, mid, late: time before gastrulation; scale bar: 50µm. (D-F) Quantification of the nuclear Med-GFP gradient from A, B and C. (G) Plots of early, mid, and late gradients from an averaged embryo (*n*=5). Error bars: s.e.m. The extents of BMP target gene Types I-III are illustrated at the bottom. (H) Time course of BMP signaling for different locations along the dorsoventral axis. s.e.m. depicted as corridors around the mean due to high density of points. Inset: illustration of embryo with nuclei colored to correspond to curves in the plot.

To measure the dynamics of nuclear Med-GFP, we imaged live embryos (*n*=5) end-on with a confocal microscope roughly 100 μm from the posterior pole (Fig. 3A-C) (Carrell and Reeves, 2015). Expression of H2A-RFP allowed us to segment the nuclei to distinguish nuclear from cytoplasmic pixels. The Med-GFP gradient was initially broad and weak, then refined into a narrow, intense peak before gastrulation (Fig. 3D-F; Movies 1-5). To model the nature of the BMP transient, especially at the location of the Type III boundary (∼25% DV), we averaged data from the embryos (see ‘Averaging multiple embryos’ section of Supplemental Materials and Methods and Movie 6). These quantitative measurements captured crucial aspects of the BMP signaling dynamics that were previously unknown, including the single-embryo dynamics of gradient amplitude and width, as well as tracking of the transient at specific DV coordinates. In particular, we found the early peak was wide enough to activate the Type III domain, yet the gradient at later time points was not (Fig. 3G). In its most refined state, the gradient was wide enough to plausibly activate genes in both the Type I and II domains.

Nuclei that lay at the boundaries of Type I-III domains experienced distinct BMP dynamics. Nuclear Med-GFP intensities at 4% DV (Type I boundary) rose continuously relative to basal levels (Fig. 3H), which represent the levels of Med-GFP in the nucleus at resting state, found on the ventral half of the embryo. Note that, as described previously (Sutherland et al., 2003), basal levels are above the zero (background) levels of GFP, which were determined by control embryos expressing H2A-RFP but not Med-GFP (see ‘Control embryos expressing H2A-RFP but not Med-GFP’ section in Supplemental Materials and Methods and Fig. S3). In contrast, nuclei between the Type II and III boundaries experienced a transient in GFP intensity. At 9% DV (Type II boundary), GFP intensity peaked roughly 15-20 mins before gastrulation, followed by a slight decrease as the gradient continued to refine. The transient intensity at the Type III boundary (25% DV) was even shorter: nuclear Med-GFP levels were above basal levels only briefly, then dropped to a level indistinguishable from basal levels roughly 20 min before gastrulation. Thus, cells beyond the Type II domain boundary experienced BMP signaling transiently, which raised the question of the fate of Type III transcription in cells between 9% and 25% DV.

### *pnr* is transcribed in cells that lack BMP signaling

**Fig. 4.**
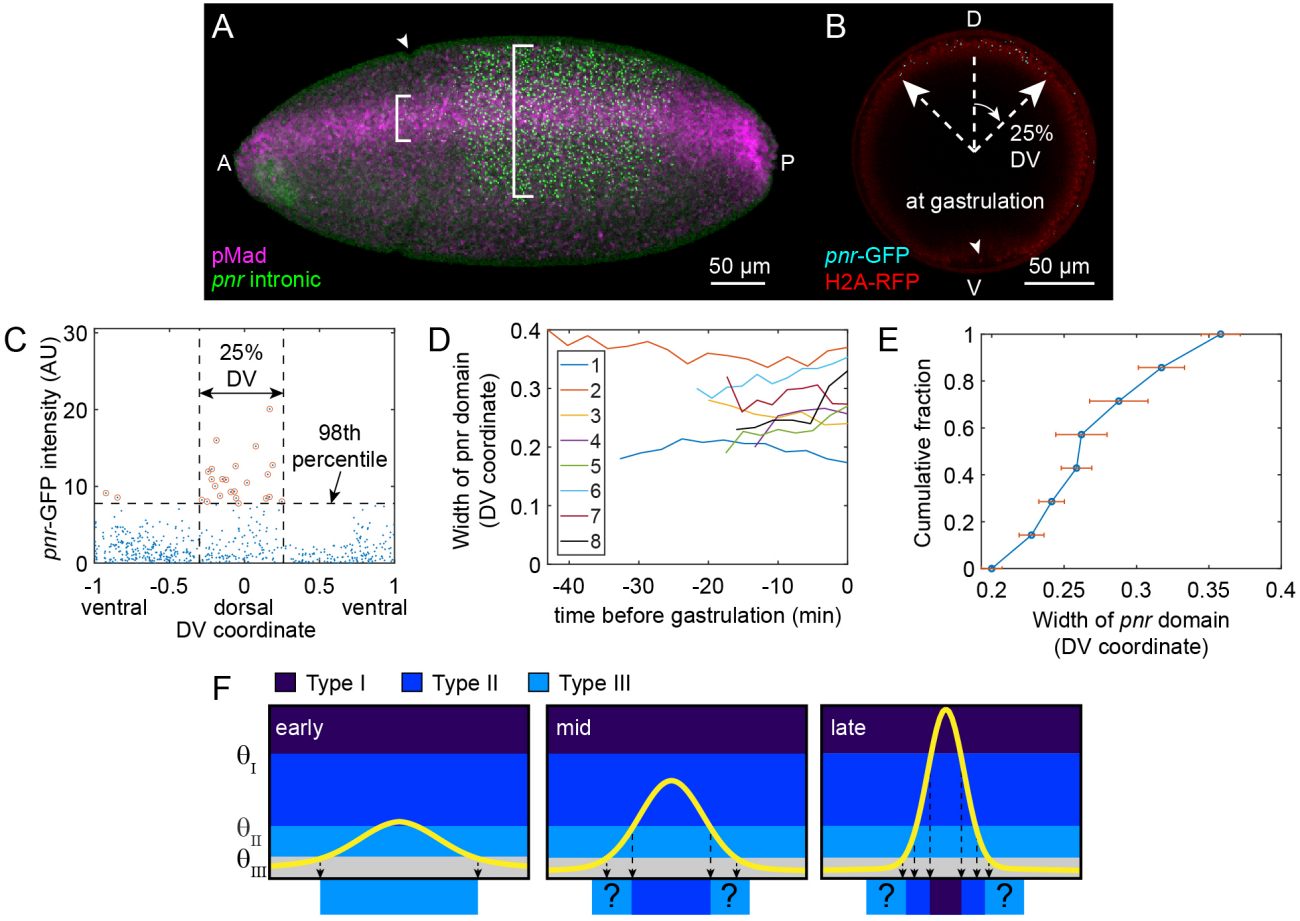
**Transcription of Type III gene *pnr*.** (A) Fixed embryo immunostained against pMad (magenta) and hybridized with an antisense probe against the first intron of *pnr* (green). At this stage, the pMad signal has refined, but the *pnr* extent remains wide (note white brackets). The cephalic furrow can be seen (arrowhead), indicating the beginning of gastrulation. (B) Max intensity z-projection of live embryo expressing the *pnr-*GFP constructs (cyan dots), as well as H2A-RFP (red). The domain of cells actively transcribing from the *pnr P3* enhancer is 25% DV. The ventral furrow can be seen at this time point (arrowhead), indicating the beginning of gastrulation. (C) Plot of the *pnr-*GFP dot intensities for all z-slices at the time point shown in (B). Dots are counted as *pnr P3* transcription if they exceed the 98th percentile. A 25% DV window captures most of these dots (see ‘Image analysis of pnr-GFP embryos’ section in Supplementary Methods). (D) Quantification of the width of the *pnr-*GFP domain from eight embryo time courses. Legend corresponds to Embryos 1-8 depicted, in order, in Movies 7-14. (E) Cumulative distribution plot of *pnr-*GFP domain widths. Points and error bars are averages and s.e.m., respectively, across each time course (*n*=8). (F) Plausible model of the relationship between BMP signaling dynamics and gene expression. Early BMP signaling is above only the threshold, θ_III_, for Type III genes. At an intermediate stage, the signal intensifies to also activate Type II genes. At late stages, the signal becomes intense enough to activate both Types I and II. However, the location where the signal surpasses the Type III threshold is much narrower than the Type III domain (see vertical down arrows). The question marks represent a putative memory mechanism.

To measure the extent and timing of Type III gene expression, we first detected nascent transcripts of *pnr* in fixed embryos using RNA probes that hybridize to the first intron of *pnr* (Fig. 4A). We co-immunostained these embryos with an antibody against pMad. In Stage 6 embryos, when the cephalic furrow was evident, the pMad stripe had become narrow and intense, while the domain of active *pnr* expression remained wide. These results suggested that a short transient of BMP signaling is sufficient to activate long-term *pnr* expression. However, the *pnr* domain is complex, comprising at least two overlapping sub-patterns: the dorsal patch and a six-striped pattern (Liang et al., 2012). While the ventral boundary of the six-striped pattern is set by the activity of the repressor Brinker (Brk) (Jaźwińska et al., 1999a,b), which is expressed in a broad lateral stripe between ∼50% and 80% DV (Fig. S4), BMP signaling sets the boundary for the dorsal patch.

To specifically measure the dynamics of expression in the BMP-dependent dorsal patch, we used the MS2 system in live embryos (Bertrand et al., 1998; Forrest and Gavis, 2003; Forrest et al., 2004; Weil et al., 2006; Garcia et al., 2013; Lucas et al., 2013). We chose to drive expression of a reporter gene, *lacZ*, by the *pnr P3* enhancer fragment, which directs expression primarily of the dorsal patch, but also includes two of the six stripes (Liang et al., 2012). In BMP mutants, the dorsal patch driven by the *P3* enhancer is lost (Liang et al., 2012). Therefore, we chose to analyze the *pnr P3* enhancer because of its specificity for BMP signaling. We placed 24× MS2 sequences in the 5'UTR of the reporter construct. For convenience, we refer to the *pnr P3-MS2-lacZ*/MCP-GFP combination as *pnr-*GFP. We imaged these live embryos (also expressing H2A-RFP) end-on, which revealed nuclear dots of nascent transcripts (transcription in progress) of *pnr*-GFP in a wide domain consistent with the Type III domain, even at gastrulation (Fig. 4B). We quantified the width of the expression domain by determining the fraction of the dorsoventral axis that contains the highest number nuclear dots above the 98th percentile of intensity while leaving out those below (Fig. 4C; see ‘Image analysis of pnr-GFP embryos’ section in Supplementary Materials and Methods for more details). After quantifying the *pnr-*GFP expression domain in multiple embryos (*n*=8; see Fig. 4D-E and Movies 4-11), we found that the domain width does not systematically change over the course of nc 14 (Fig. 4D). These data show that the *pnr* dorsal patch maintains active gene expression in the Type III broad domain (27% ±5% DV; Fig. 4E) until gastrulation begins. The transient nature of BMP signaling in these cells, together with the continued expression of *pnr*, could plausibly imply that some Type III genes require BMP signaling for activation, but not maintenance, which may constitute a memory, or ‘ratchet’ mechanism (Fig. 4F).

It has been shown that other factors regulate *pnr* expression (Liang et al., 2012). In particular, Brk at least partially sets the boundary of *pnr*; however, there is also a portion of *pnr* expression that (1) requires BMP for activation (Winick et al., 1993), and (2) is not significantly regulated by endogenously expressed Brk. Our results suggest that, at a minimum, the dorsal patch, largely driven by the *pnr P3* enhancer, is regulated by a memory mechanism. While it is unclear what constitutes the proposed memory mechanism, it could be transcriptional feedback, such as self-activation of *pnr*, or perhaps based on chromatin states.

This mechanism, in which the precise BMP dynamics are responsible for the proper pattern, could be similar to Hedgehog (Hh) signaling in the *Drosophila* wing disc. In that system, Hh signaling initially expands across ∼10 cell widths (Strigini and Cohen, 1997; Nahmad and Stathopoulos, 2009), but later refines due to a feedback mechanism. The cells that receive transient exposure of Hh signaling are able to permanently activate *dpp* expression. On the other hand, the receptor *patched* requires sustained Hh signaling, and is thus expressed in a narrow domain (Nahmad and Stathopoulos, 2009). Furthermore, a ratchet mechanism has recently been proposed for Dorsal-dependent activation of Twist on the ventral side of the embryos (Irizarry et al., 2020). Given these additional cases in which a memory mechanism may play a direct part in patterning, we propose this mechanism may be more widespread than previously appreciated.

## MATERIALS AND METHODS

### Fly lines

The following fly lines were obtained from the Bloomington Drosophila Stock Center: His2AV-RFP (w[*]; P{w[+mC]=His2Av-mRFP1}II.2 -- BS# 23651), His2AV-RFP; MCP-GFP (y[1] w[*]; P{w[+mC]=His2Av-mRFP1}II.2; P{w[+mC]=nos-MCP.EGFP}2 -- BS# 60340), *nos*-Gal4 (w[1118]; P{w[+mC]=GAL4::VP16-nos.UTR}CG6325[MVD1] – BS# 4937). The UAS-Med-GFP line was a kind gift from Laurel Raftery (University of Nevada, NV, USA) (Miles et al., 2008). Wildtype flies were *yw* mutants obtained from the Stathopoulos lab (McMahon et al., 2008).

### Creation of pnr P3-24× MS2-lacZ construct

The Pnr-P3 enhancer region was amplified from genomic DNA of wild-type flies using forward primer ACGGACGGCAGTCATTAACA and reverse primer GCTTTTATGGCCCCTACGGA. These sequences were cloned into the injection vector piB-hbP2-P2P-MS2-24x-lacZ-αTub3′UTR (a kind gift from Thomas Gregor, Princeton University, NJ, USA) (Garcia et al., 2013) by replacing the hbP2 enhancer with the pnr p3 enhancer and the hb P2 promoter with an eve minimal promoter. The eve minimal promoter was amplified using forward primer GAGCGCAGCGGTATAAAAGG and reverse primer GGTCCACGGGACTGGCGTCG. These regulatory sequences in the plasmid drove the expression of lacZ, which contained 24 copies of the MS2 stem loops in its 5′UTR. This vector was injected into the 38F1 fly line (Genetivision) for recombination-mediated cassette exchange (RMCE) (Bateman et al., 2006).

### Fixation and immunostaining of embryos

Wild-type (laboratory strain yw) or nos-Gal4/UAS-Med-GFP embryos were aged to 2-4 h after egg lay, then fixed in 37% formaldehyde according to published protocols (Kosman et al., 2004). Fluorescent *in situ* hybridization and fluorescent immunostaining were performed according to published protocols (Kosman et al., 2004).

Primary antibodies used were rabbit anti-Human Phospho-Smad2/3 (R&D Systems MAB8935, 1:500 dilution), goat anti-histone (Abcam ab12079, 1:100 dilution), goat anti-GFP (Rockland 600-101-215, 1:400 dilution), and goat anti-fluorescein (Rockland 600-101-096, 1:500 dilution; to detect the *pnr* intronic probe). In embryos co-stained for pMad and GFP, nuclei were detected with DAPI (Vectashield H-1200).

Secondary antibodies used were donkey anti-rabbit-Alexa Fluor 546 (Invitrogen A10040, 1:500 dilution) and donkey anti-goat conjugated with Alexa Fluor 647 (Invitrogen A21447, 1:500 dilution).

### Imaging fixed embryos

Nc 14 embryos expressing Med-GFP and immunostained against pMad and GFP were cross sectioned using a razor by removing the anterior and posterior thirds of the embryo, as described previously (Carrell and Reeves, 2015). The cross sections were mounted in 70% glycerol and imaged using a Zeiss LSM 710 inverted microscope with photomultiplier tube detectors at 20× magnification. The embryos were imaged with a pixel dwell time of 6 μs, a pinhole size of 30 μm, and a band pass filter between 493 and 550 nm for the green channel and between 572 and 630 nm for the red channel. Images were taken as 512×512 images in a 16 bit format. Z-stacks were taken with 15 z-slices at 1.5 μm intervals.

### Imaging Med-GFP in live embryos

To image Med-GFP, *yw*; *His2AV-RFP*; *nos-Gal4* flies were crossed to *UAS-Med-GFP* flies. F1 females carrying all three constructs were then placed in embryo collection cages with males. The progeny embryos were collected for 1 h on grape juice agar plates, dechorionated using 100% bleach (7.5% sodium hypochlorite) for 30 s, then rinsed using deionized (DI) water. To prevent drying during mounting, the embryos were placed onto an agar plate. These embryos were then mounted vertically to capture optical cross sections.

There were two sets of embryos; the first set (two of the five embryos) were mounted and imaged in the following way. First, they were transferred to the short edge of a 22 mm coverslip, to which heptane glue (Supatto et al., 2009) had been applied, as described previously (Carrell and Reeves, 2015). Briefly, each embryo was oriented perpendicular to the short edge of the coverslip, such that approximately half of the embryo was adhered to the heptane glue on the coverslip and half was hanging off. The coverslip was then adhered with double-sided tape to a 21.5 mm-tall steel mounting block in the shape of a half cylinder with a 30 mm diameter (Carrell and Reeves, 2015). The short edge of the coverslip to which embryos were not adhered was aligned with the top of the mounting block, such that 0.5 mm of the coverslip (to which embryos were adhered) was extending past the bottom of the mounting block (Carrell and Reeves, 2015). The mounting block was then placed into a glass-bottom 35 mm Petri dish with number 1.5 coverslip (Mattek P35G-1.5-14-C), centered such that the 0.5 mm overhang of the coverslip was inside the cut bottom of the Petri dish. DI water was added to the dish to prevent embryos from drying out. The glass-bottomed dish/mounting block was placed on the Zeiss LSM 710 inverted microscope with photomultiplier tube detectors to collect images. A 40×1.1 numerical aperture (NA) water immersion lens with 0.6 mm working distance was used. The long working distance allowed for imaging of single z-slice optical cross sections of the embryos >100 μm away from the coverslip. The mounting process, including dechorionization, took roughly 1 h so that embryos were at or near nc 14 upon the beginning of imaging. The embryos were imaged with a pixel dwell time of 2 μs, a pinhole size of 78 μm, and a band pass filter between 500 and 561 nm for the green channel and between 561 and 632 nm for the red channel. A time resolution of 25 s between frames was used. Images were taken as 1024×1024 images in a 16 bit format.

The second set of embryos (three of the five embryos) were mounted and imaged in the following way. The embryos were transferred to glass-bottomed Petri dishes with number 1.5 coverglass (Mattek P35G-1.5-20-C). Each embryo was mounted in 10 μl of 1% low melt agarose (IBI Scientific IB70051). A hair loop, fixed by tape to a pipet tip, was used to orient the embryos perpendicular to the coverglass and to adhere the anterior tip to the coverglass. After the agarose gelated, deionized water was added to the dish to prevent desiccation, as described previously. The glass-bottomed dishes were then placed on Zeiss LSM 900 inverted microscope with GaAsP photomultiplier tube detectors to capture images. A 40×1.2 NA water immersion lens was used to obtain single z-slice optical cross sections of the embryos 100 μm away from the anterior pole. This mounting process, not including dechorionization, took approximately 5 min per embryo. The embryos were imaged with a pixel dwell time of 2 μs and a pinhole size of 78 μm. The green channel light was sent to the detector with a dichroic that reflected light greater than 509 nm, and a band pass filter between 400 and 575 nm was used. For the red channel, a band pass filter between 570 and 700 nm was used. A time resolution of 45 s between frames was used. Images were taken as 1024×1024 images in a 16 bit format.

### Imaging pnr-GFP in live embryos

To image *pnr*-GFP, virgin females of yw; His2AV-RFP; MCP-GFP flies were placed in embryo collection cages with males with *pnr-P3* enhancer 24xMS2-*lacZ* construct. The progeny embryos were subject to the same mounting and imaging procedure as the described in the first set of Med-GFP embryos, with the following exceptions: The embryos were imaged with a pixel dwell time of 6 μs, a pinhole size of 40 μm, and a band pass filter between 493 and 551 nm for the green channel and between 601 and 690 nm for the red channel. Furthermore, z-stacks between 10-21 slices, 2 μm per slice were taken, with each z-stack starting at a distance of 75 μm from the posterior pole. Movies and images of *pnr*-GFP embryos were created as max-intensity projections of the z-stack.

### Image analysis of cross-sections of embryos

The analysis of the embryo images was conducted in accordance with the previously published protocols (Trisnadi et al., 2013). The image analysis involves three crucial steps: (1) unrolling the embryo, (2) segmenting the nuclei, and (3) empirically fitting the intensities. Detailed descriptions of these steps are provided below.

### Unrolling the embryo

To facilitate the nuclear segmentation process in both live and fixed embryos, we computationally unrolled the cross sections of the embryos resulting in a 2D strip. To unroll the embryo, we followed our previously published protocols (1). Briefly, we first filtered the image with a 2D Gaussian filter with a five-pixel radius. Next, we found the border of the embryo in the filtered image. Then, using the detected boundary as reference, we found an ‘inner border’ that was roughly 18.36 μm interior to the outer border (a liberal estimate of the average apical–basal height of the nuclei). Thus, between the outer and inner boundaries, we formed an annulus (ring) which contained the nuclear layer.

Once we had the coordinates for the outer and inner boundary, we used projective transformation to unroll the ring-shaped nuclear strip to a rectangular strip (see Fig. S6B). After the nuclei are segmented in the unrolled strip, the nuclear mask is transformed back into the original image coordinates for further processing (see below).

The unrolling method, as well as all computational methods, were performed with Matlab.

### Segmentation of nuclei

Once we transformed the nuclear layer of the embryo cross section into an unrolled strip, we segmented the nuclei in the following manner. First, we calculated the regional maxima for the unrolled strip using Matlab's ‘imregionalmax’ function, and these maxima were used as foreground markers for the marker-based watershed (see  Fig. S6C). This step also detected the intensity maxima for the nuclei and some parts of the cytoplasm (due to noise) as well. We kept the spurious maxima that were due to noise in the cytoplasm as it allowed us to get a tighter fit for the nuclei in the next step.

Using the watershed technique allowed us to obtain ridge lines (watershed lines) that separated each nucleus into individual compartments (Fig. S6D). This was effective as we were then able to split each nucleus to its own territory and could process each nucleus without affecting others. Using this approach also alleviated issues where global approaches to segmenting nuclei would not have worked due to variation in intensity among the nuclei.

Once we had the individual compartments, we discarded the compartments that contained only cytoplasm. We identified which compartments contained nuclei by iterating through each vertical line (1 pixel thick) of the original unrolled strip and noting the coordinates where there is sudden increase or drop in intensity. These coordinates were where the nuclei began and ended. Using these coordinates, we generated a smoothened outline for the area occupied by the nuclei.

Next, for each compartment that contains a nucleus, Otsu's method was used for thresholding to obtain a binary mask. Note that each compartment was subject to a different threshold value due to variations in intensity across the image, which precluded using a global threshold value (Movie 12 illustrates the binary mask estimation for each compartment of a single unrolled strip). Once a complete binary mask was determined for the entire unrolled strip, it was then cleaned by morphological opening and removing large components. In addition to cleaning, we also eliminated artifacts by thresholding components below 400 pixels in size (see Fig. S6F, S6G).

This cleaned binary mask served as a nuclear mask that outlined the border of individual nuclei. In addition to the nuclear mask, average histone-RFP intensity and centroid location were also noted. This mask was then transformed to the original coordinates using the earlier transformation to align with the original image of the embryo (Fig. S6H). See Fig. S5 for the pipeline used for image analysis and Fig. S6 for the image results obtained at the corresponding intermediate stages.

### Intensity analysis of BMP gradients in live and fixed embryos

The mean intensity and standard error of the mean intensity of the pMad channel (Fig. 2) and Med-GFP channel (Figs 2,3) were recorded. The position of each nucleus along the dorsoventral axis was also recorded.

### Empirical fitting of BMP gradients in live and fixed embryos

Given the locations of centroids of the nuclei and their perimeters, we extracted the intensities of the nuclei in the nuclear channel (histone-RFP for live embryos and DAPI for fixed) and the Med-GFP channel (and the pMad channel in fixed embryos). This information was then used to calculate the mean and standard deviation of the intensities of all channels in each nucleus. The nuclear channel intensity was in part used to normalize for uneven intensities in the Med-GFP or pMad channel (Trisnadi et al., 2013; Liberman et al., 2009; Reeves et al., 2012) (1–3). In fixed embryos, the intensity of the Med-GFP or pMad channel in nucleus *i* was directly normalized by the corresponding intensity of the nuclear channel in nucleus *i* (1). The normalization procedure for live embryos is given below. Next, we fit the normalized intensity data for each nucleus in the Med-GFP or pMad channel to Gaussian shaped curves described by:

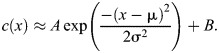


In the equation above, the four parameters that were estimated by fitting the intensity values for each time frame are:

A, amplitude; B, basal levels; μ, location of the presumptive ventral midline; and*σ*_,_ length scale of the gradient or the gradient width. A non-linear least squares approach was used to optimize the fit for the gradient curves. In fixed embryos, the gradients were normalized by subtracting the basal levels and dividing by the amplitude (Fig. 2D of main text). For live embryos, the fit of the Med-GFP gradient for each frame was treated independently and resulted in a time course for the amplitude, basal levels, and gradient width [*A*(*t*), *B*(*t*), *σ*(*t*), respectively].

### Normalization across the dorsoventral axis, different embryos, and imaging sessions for live Med-GFP embryos

To be able to compare nuclear Med-GFP intensities across the dorsoventral axis, from embryo to embryo, and across multiple imaging sessions, we performed several calibration steps. First, to normalize across multiple imaging sessions, we measured the laser intensity before the start of each imaging session by sending the laser, without sample, into the transmitted light detector to create a calibration image (Trisnadi et al., 2013; Al Asafen et al., 2020). The average intensity of the calibration image was stored as a variable called *Calib*_*mean*_. In addition, for this calibration measurement, the laser power setting on the Zen software, *Calib*_*LP*_, was noted. We used these two measurements to create a calibration normalization, *Calib*_*norm*_:

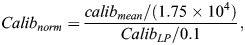
where the two constants 1.74×10^4^ and 0.1 were chosen to ensure *Calib*_*norm*_ was close to 1. The Med-GFP channel from each embryo in an imaging session was normalized by the corresponding *Calib*_*norm*_.

Next, to normalize for embryo-to-embryo variation, the mean nuclear (Histone-RFP) intensity across the entire time course, *nuc*_*mean*_, was calculated. The laser power for the RFP channel, *nuc*_*LP*_, was also noted. We used these two measurements to create a time course normalization, *timecourse*_*norm*_:

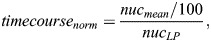


where the constant 100 was chosen to ensure *timecourse*_*norm*_ was close to 1. The Med-GFP channel from each embryo was normalized by its corresponding *timecourse*_*norm*_. The goal of this normalization was to corrects for an overall brighter time course in the nuclei. This could be an issue if, for example, one time course was taken at z=160 μm from the pole and another at z=140 μm. The overall nuclear intensity should be the same across every embryo imaged.

Finally, to normalize for variations in intensity across the dorsoventral axis, we created a normalization curve as a function of DV coordinate for each embryo based on the nuclear intensity. To do this, for each frame *j*, we correlated the mean intensity of Histone-RFP in each nucleus with the DV coordinate and smoothed this correlation with a sliding window of 30 nuclei. This smoothed correlation was then linearly interpolated onto an equally spaced grid of 151 points, *x*_*grid*_, which resulted in *curve*_*norm*,*j*_(*x*_*grid*_). A final normalization curve for the whole time course, 
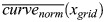
, was then produced by averaging *curve*_*norm*,*j*_(*x*_*grid*_) across all frames. It was assumed that variations in intensity of the nuclei across the dorsoventral axis were constant across the entire time course, possibly due to uneven paths to and from the objective. To normalize the nuclear intensities of Med-GFP in frame *j*, 
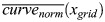
 was re-interpolated onto the locations of the individual nuclei, *x*_*nuc*_, in frame *j* to give 
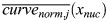
.

Once we had these normalizations, we performed the following transformations to the data to ensure day-to-day, DV axis, and frame-to-frame normalization:

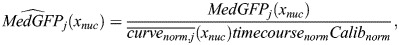
where 
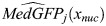
 is the normalized Med-GFP intensity in frame *j* as a function of *x*_*nuc*_.

Two of the live Med-GFP embryos were taken on a Zeiss LSM 710 confocal microscope, and three were with a Zeiss LSM 900. Each set of embryos also had a corresponding set of control embryos, which expressed H2A-RFP without Med-GFP. Because of the differences in microscopes, the two sets of embryos needed to be normalized in order to be compared. We normalized by the average green-channel intensity of the respective control embryos (see ‘Control embryos expressing H2A-RFP but not Med-GFP’ section).

### Averaging multiple live Med-GFP embryos

To reduce the noise in the gradient measurements, we used the Med-GFP live embryos to create an average gradient. To do this, it would not be appropriate to average the gradients simply directly from the embryos together, for two reasons. First, the embryos had different gradient widths, but similar shapes, so averaging them together would result in an intermediate width gradient but would alter the shape. And second, the gradients were composed of nuclei with DV coordinates that did not match a well-spaced mesh. Therefore, we first rescaled the gradients to conform them to an average gradient width, then normalized them by subtracting their basal levels and dividing by their amplitude. Then, after binning them together, we ‘de-normalized’ them by multiplying by the average amplitude and adding the average basal levels. The final step was to bin and interpolate the nuclei onto a regular mesh. Details for these processes are given below.

The first step was to find average gradient parameters. Therefore, for each embryo *k*, we smoothed the amplitude, basal levels, and gradient width in time with a sliding window of 10 frames to give *A*_*k*_(*t*), *B*_*k*_(*t*), *σ*_*k*_(*t*), respectively. Then we averaged them together over all embryos to give 
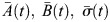
.

The next step was to conform the gradients to an average gradient width. To do this, for each frame of each embryo *k*, we rescaled the DV coordinate by multiplying by 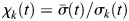. This resulted in the embryos’ gradients either ‘stretching’ or ‘compressing’, depending on whether *χ*_*k*_(*t*)>1 or < 1, respectively. Nuclei with rescaled DV coordinate values >1 or <−1 were ignored, which did not pose a problem, because near the ventral midline, the gradient had already decayed to zero.

Next, for each embryo *k* and each frame *j*, the Med-GFP intensity in each nucleus, normalized as described in the previous section, was further normalized by subtracting the basal levels and dividing by the amplitude. Then, for the corresponding frames in each embryo, we binned together the further normalized intensities and de-normalized them by multiplying by 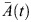 and adding 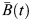. We then combined the right and left halves of the dorsoventral axis of this ‘binned embryo’ so that all nuclei had DV coordinates between zero and one.

The final step to create an averaged gradient was to place it onto a well-spaced mesh. This is because the ‘binned embryo’ intensity gradient was still composed of nuclei with DV coordinates that did not match a well-spaced mesh. To do this step, a mesh was created, *x*_*mesh*_, from zero to one, with 36 points, resulting in a spacing of *dx*=0.0286. For all *i*=2 to 35, the intensities of nuclei with coordinates greater than 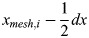 and less than 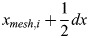 were averaged together. For *i*=1, the intensities of nuclei with coordinates between zero and 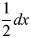 were averaged together, and for *i*=36, the intensities of nuclei with coordinates between 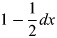 and 1 were averaged together. Some bins were empty (had no nuclei); the gradient at these points were interpolated between neighboring bins. Error bars at each point were the standard error of the points in each bin and interpolated for empty bins.

### Control embryos expressing H2A-RFP but not Med-GFP

Embryos expressing H2A-RFP but not Med-GFP were imaged using the same imaging protocols as the Med-GFP embryos, with a first set of control embryos taken on the Zeiss LSM 710, and a second set on the Zeiss LSM 900. The embryos were analyzed using the same image analysis methods as the Med-GFP embryos, including the procedures to account for different embryos and different imaging sessions. This analysis resulted in intensity values from the GFP channel for each segmented nucleus and for each time frame. However, because there were no markers to distinguish between the dorsal and ventral side, we averaged the GFP channel intensity for the whole dorsoventral axis, resulting in intensity curves as a function of time but with no spatial variation (Fig. S3A). We found the curves for the first set of control embryos had unexplained oscillatory intensity variations of a period of roughly 9 min. Therefore, we applied a low pass filter to average out these intensity fluctuations (compare blue versus red curves in Fig. S3B-E). For comparison, we plotted the four filtered curves for the first set of control embryos in Fig. S3F, and the four curves for the second set of control embryos in Fig. S3G.

As explained above, because the first set of control embryos were taken on a Zeiss LSM 710 confocal microscope, and the second set on a Zeiss LSM 900, the two sets of control embryos needed to be normalized in order to be compared. We chose to normalize by each set of control embryos by the average intensity within the set. In other words, we calculated *β*_*norm*,1_, the normalization constant for the first set of control embryos, as the average of all embryos in the first set, and also average over all time points. Similarly, *β*_*norm*,2_ was the normalization constant corresponding to the average of the second set of control embryos. After each set was normalized by its own normalization constant, we plotted all background curves on top of each other in Fig. S3H (black curve=average ±s.e.m.).

The basal levels for the embryos expressing Med-GFP were above the background levels from the control embryos, suggesting that, even in a resting state, some Med-GFP is in the nucleus. To see this, after normalization of the basal levels by *β*_*norm*,1_ (for the first set of Med-GFP embryos) or *β*_*norm*,2_ (for the second set), we plotted all basal levels together in Fig. S3I (red curves; average in blue). The average normalized background levels are plotted as the black curve with error bars representing the s.e.m.

### Spot detection and analysis in live pnr-GFP embryos

To analyze the pnr-GFP embryo images we first detected the spots of high intensity as described in Trisnadi et al. (2013). Briefly, for each time frame and z-slice, the nuclei were segmented as described above and the histone-RFP intensities (*I*_*nuc*_) were recorded. Then, to determine whether a nucleus contained a spot corresponding to a legitimate cluster of nascent transcripts, the following procedure was followed. First, for each nucleus, the max intensity pixel in the GFP channel was found, and its intensity (*I*_*max*_) was recorded. Second, a 5×5 pixel neighborhood, centered on the max intensity pixel, was removed from the nucleus, and the mean GFP intensity of the rest of the nucleus (*I*_*mean*_) was recorded. Therefore, each nucleus in the z-stack time course received a score *S*=(*I*_*max*_−*I*_*mean*_)/*I*_*nuc*_. For each time point, nuclei with scores in the 98th percentile (threshold *θ*=0.98) or greater clustered on the dorsal ∼30% of the embryo (Fig. 4) were considered to contain nascent transcripts (Fig. 4C).

Next, we determined the border of the region of the embryo where pnr-GFP was expressed. To detect the boundaries of the domain in which cells are actively transcribing pnr based on the stochastic presence or absence of nascent transcripts, the following protocol was used. First, the approximate dorsal midline was found manually for the entire z-stack time course by locating the region of the embryo where the highest density of nascent transcripts was found. Note that finding the precise location of the dorsal midline was not crucial, as will become clear below. Next, for each time frame, the dorsoventral axis was split into left and right halves, and based on the centroid location, each nucleus in the z-stack was assigned a DV coordinate value, with *x*=0 being the dorsal midline and *x*=1 being the ventral midline. Next, for each half of the embryo (the left and right halves were treated independently), and for each location *x*_0_ along the dorsoventral axis, a score was assigned:

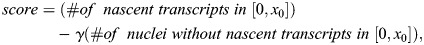


where the penalty weight was *γ*=1−*θ*=0.02. In other words, the score was equal to the number of nuclei dorsal to *x*_0_ with nascent transcripts minus 0.02 times the number of nuclei dorsal to *x*_0_ that did not have a nascent transcript. Therefore, a score was improved if there were many nascent transcripts inside the domain between the dorsal midline and the putative boundary *x*_0_, but penalized (with weight *γ*) for non-transcript-bearing nuclei inside the domain. The point of this metric was to capture a broad domain on the dorsal side that had a high percentage of nuclei with nascent transcripts while at the same time not superfluously extending the domain to include nuclei far away from the dorsal cluster that were outliers. After scoring each position along the dorsoventral axis, the boundary was considered to be the location with the highest score. After producing a score for both halves of the embryo (Fig. 4C), the width of the *pnr* boundary was considered to be the average of the boundaries of the two halves, and therefore, the exact location of the dorsal midline was immaterial. This procedure was repeated for each time point in the z-stack time course (Fig. 4D). The average width of *pnr*-GFP for an embryo's entire time course was computed as the mean of all time points in the time course, and the error bars were computed as the standard deviation of all time points in the time course (Fig. 4E).

## Supplementary Material

10.1242/biolopen.061646_sup1 Supplementary information
