# Supplementary material for: Dynamics of BMP signaling and stable gene expression in the early Drosophila embryo
Source: Biol Open. 2024 Aug 29;13(9):bio061646. doi: 10.1242/bio.061646 (PMC11381920; doi:10.1242/bio.061646)
Supplement: Supplementary information [file biolopen-13-061646-s1.pdf]

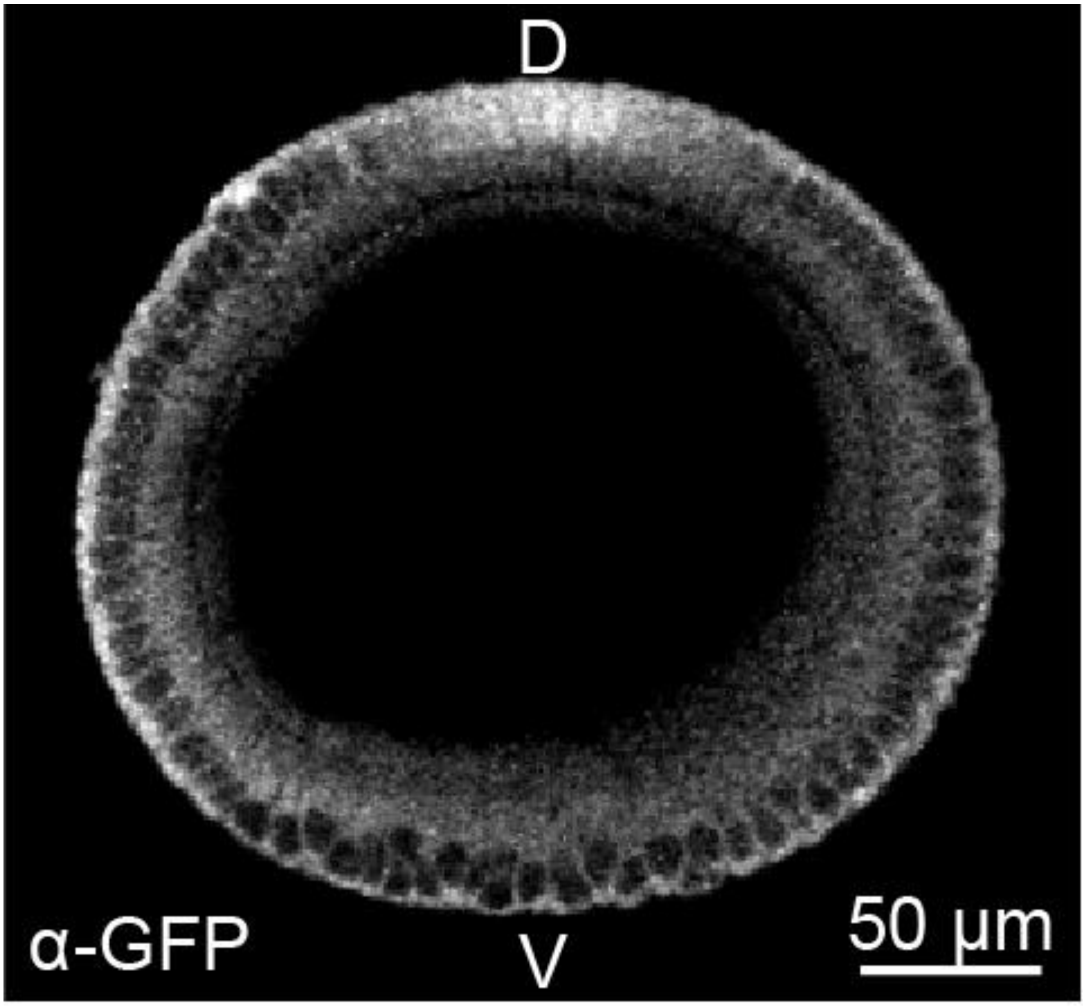

Fig. S1. Expression of Med-GFP in a gastrulation-stage embryo. This image shows that Med-GFP becomes concentrated at the site of pMad activation by the time gastrulation begins.

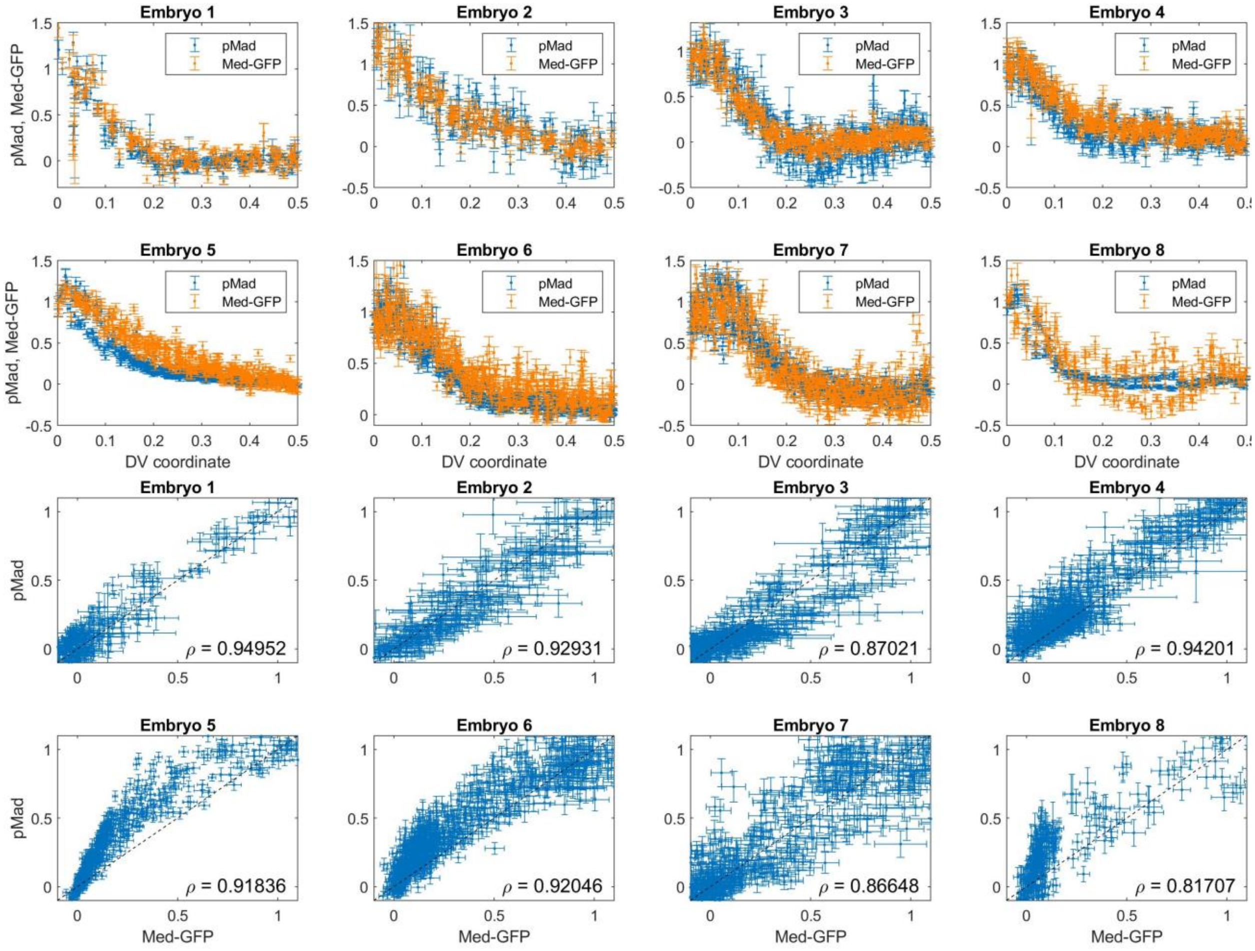

Fig. S2. Correlation between nuclear pMad and nuclear Med-GFP. Among eight embryos tested, there is a high correlation between nuclear pMad intensity and nuclear Med-GFP intensity. In the top two rows, both nuclear pMad and nuclear Med-GFP are plotted vs. DV coordinate ($x = 0$ is the dorsal midline). In the bottom two rows, nuclear Med-GFP is plotted against nuclear pMad. Correlation coefficients are shown in the bottom right. Errorbars are SEM for the intensities in each nucleus. All intensities are normalized by subtracting basal levels and dividing by the amplitude.

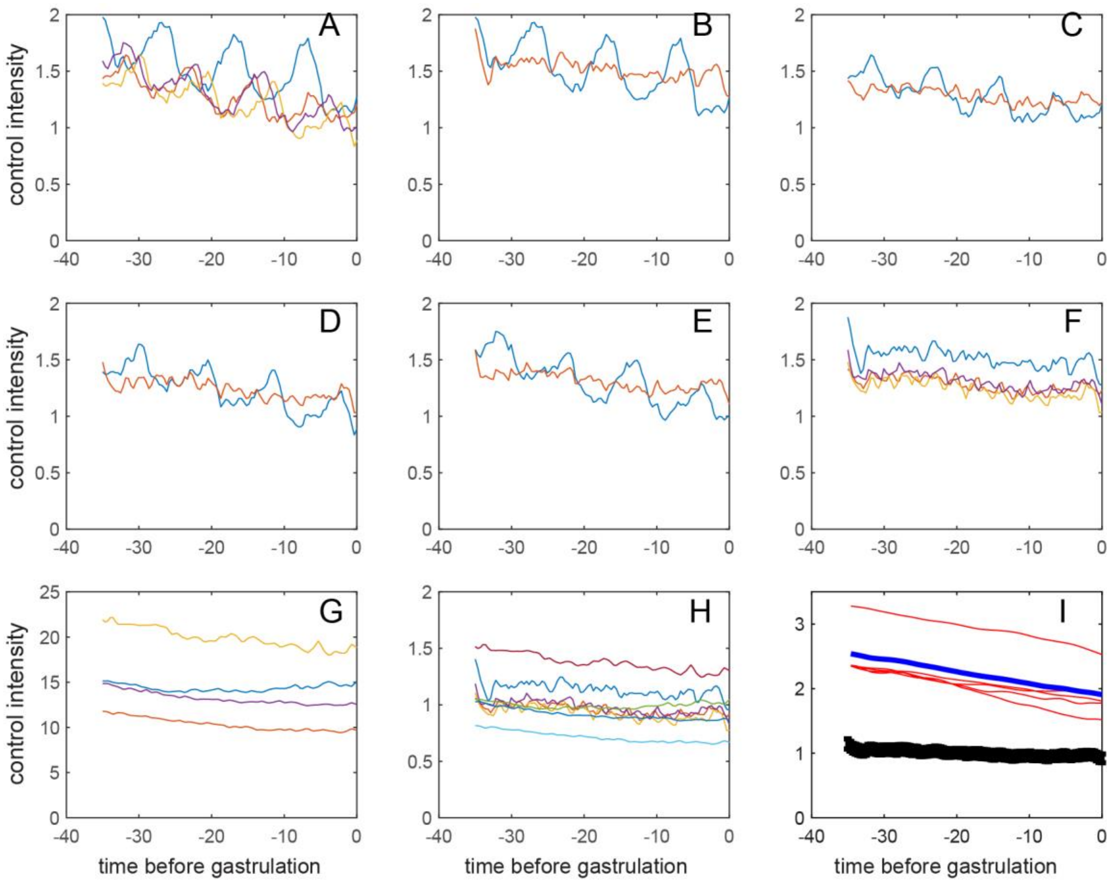

Fig. S3. Control embryos expressing H2A-RFP but not Med-GFP. (A) Intensities of the GFP channel, averaged across the DV axis, for the first set of control embryos (n = 4) as a function of time. **(B-E)** Plots of each of the four first-set control embryo average intensity curves (blue) and the intensity curves after applying a low pass filter to remove unexplained long-term oscillations. **(F)** The four filtered curves from (B-E). **(G)** The four curves from the second set of background control embryos. Note the different scale of the intensity axis. **(H)** All eight control embryos after normalization. **(I)** Normalized basal levels from all five Med-GFP embryos (red), including their average (blue curve), plotted with the average background ± S.E.M.

Biology Open • Supplementary information

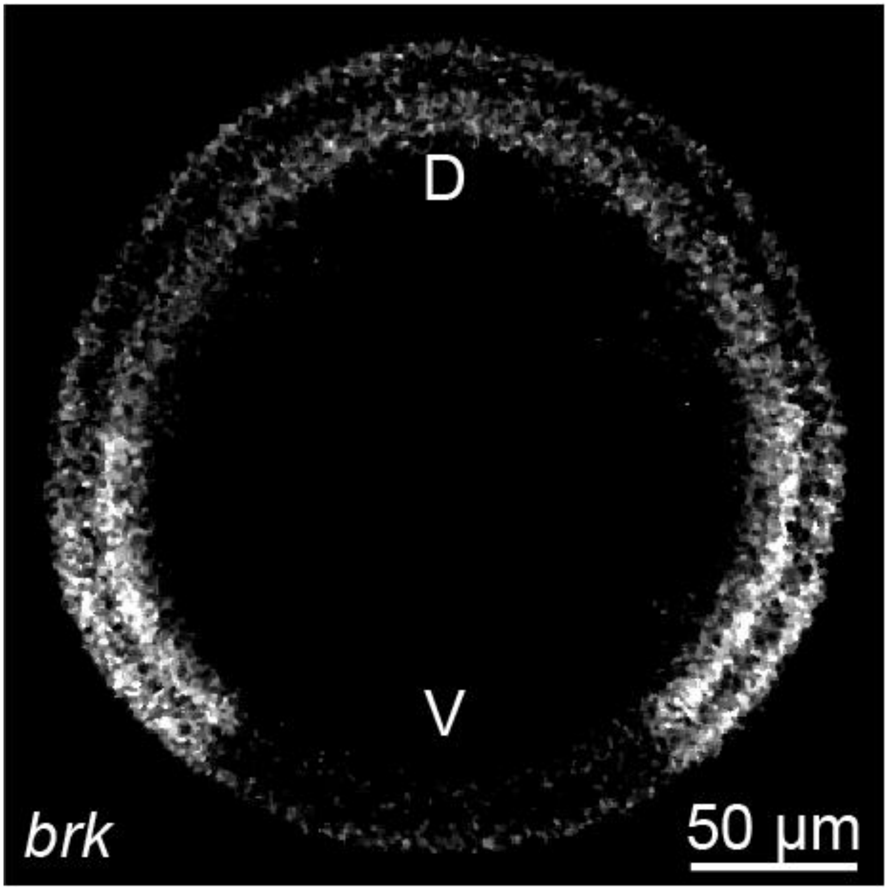

Fig. S4. Expression pattern of *brk* in nc 14 embryo. The *brk* expression pattern does not extend into the dorsal half of the embryo, which precludes it from having a cell autonomous effect on the dorsal patch of *pnr* expression in the 22-32% dorsal-most cells.

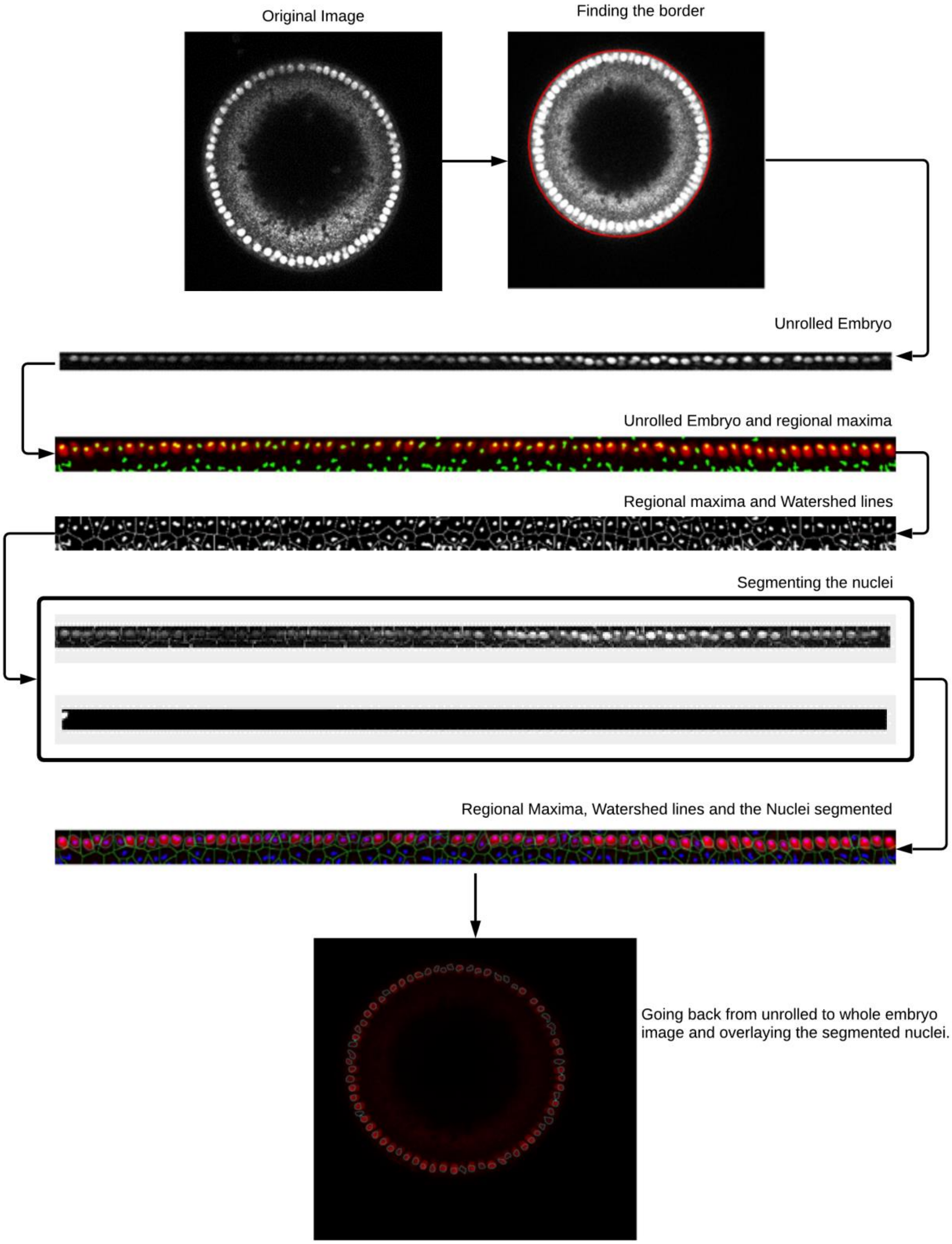

Fig. S5. Procedure to segment nuclei.

Biology Open • Supplementary information

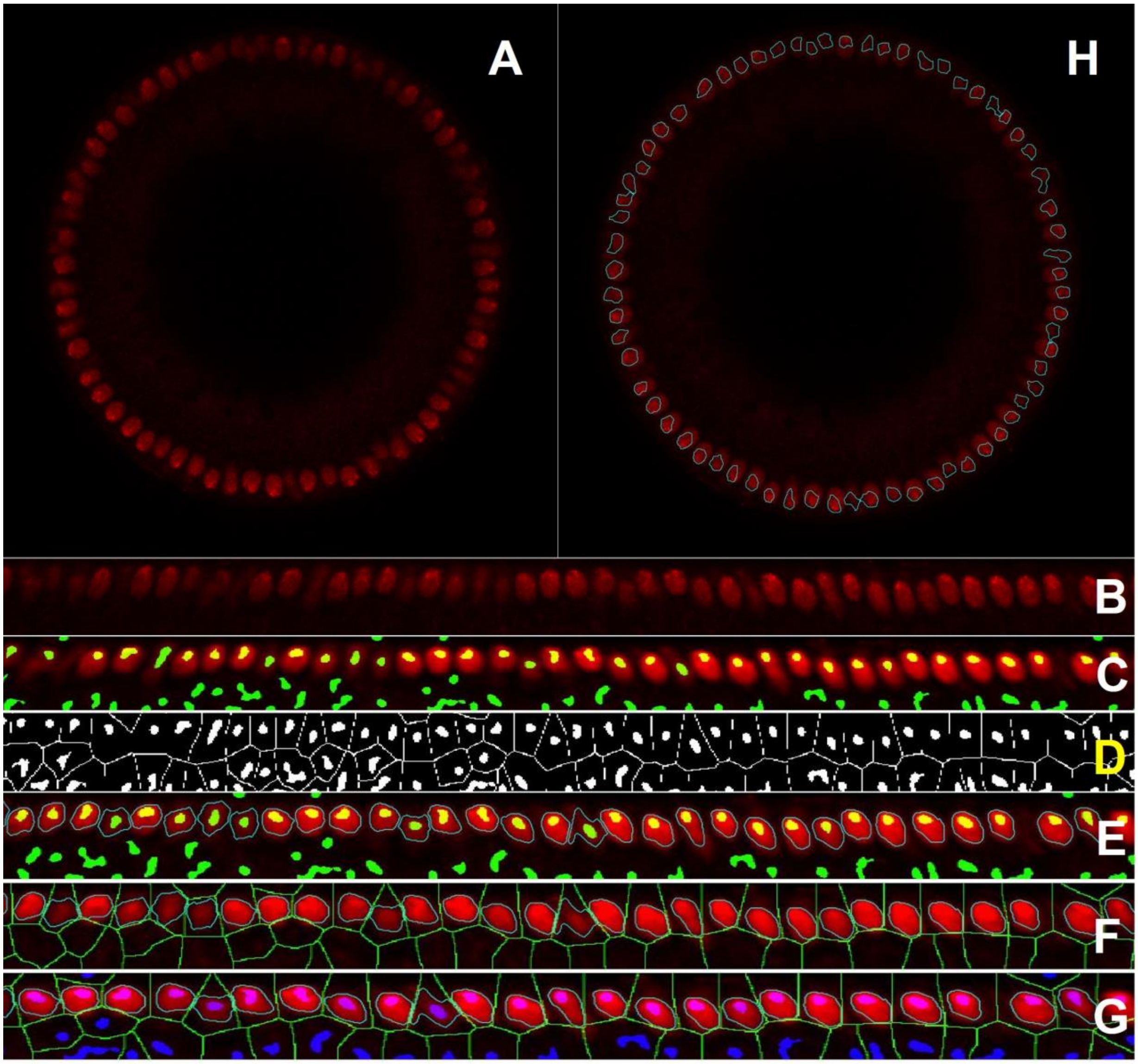

A – Whole embryo

B – Unrolled embryo

C – Nuclei and Maxima

D – Ridge liens and maxima

E – Nuclei, maxima and outline

F – Nuclei, Ridgle lines and outline

G – Nuclei, Ridgle lines, maxima and outline

H – Mask outline on whole embryo

Fig. S6. Intermediate results for nuclei segmentation.

Biology Open • Supplementary information

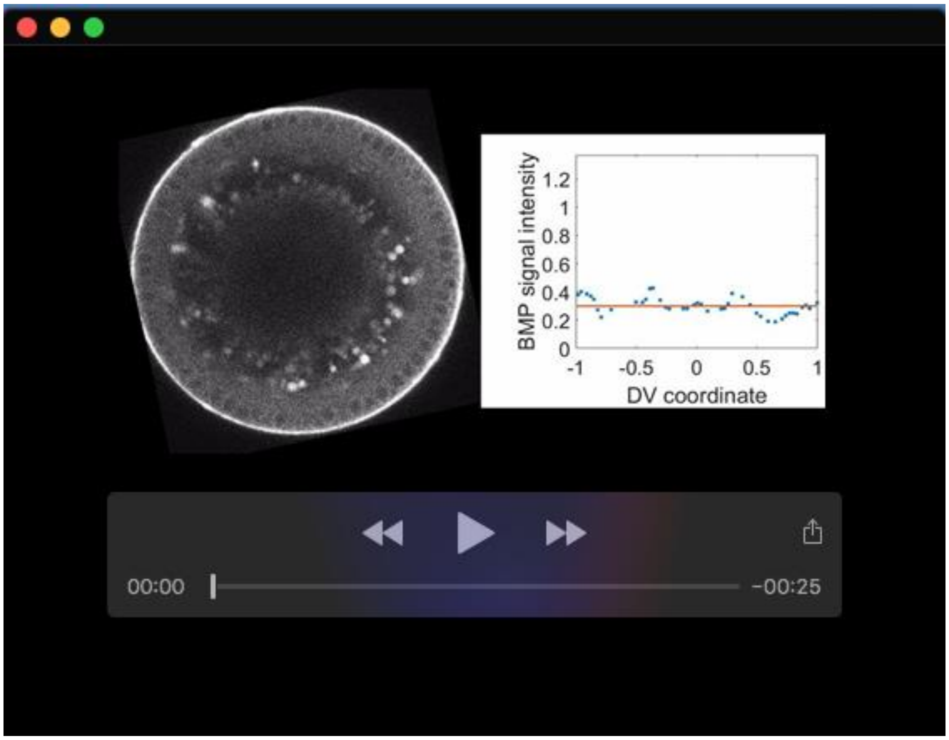

Movie 1. Time course and quantification of Med-GFP Embryos 1-5.

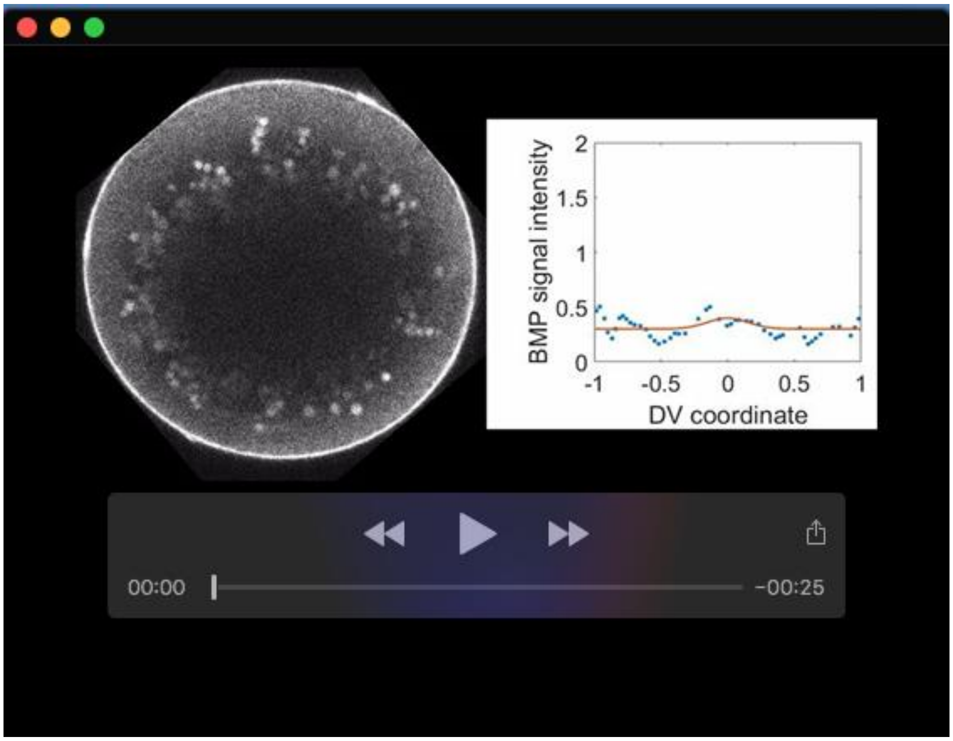

Movie 2. Time course and quantification of Med-GFP Embryos 1-5.

Biology Open • Supplementary information

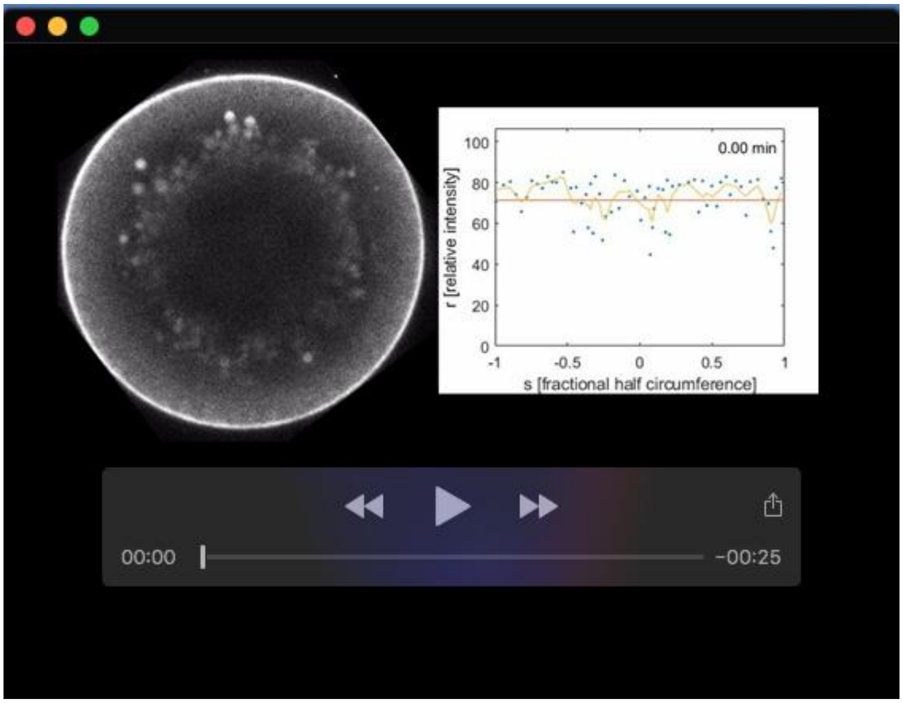

Movie 3. Time course and quantification of Med-GFP Embryos 1-5.

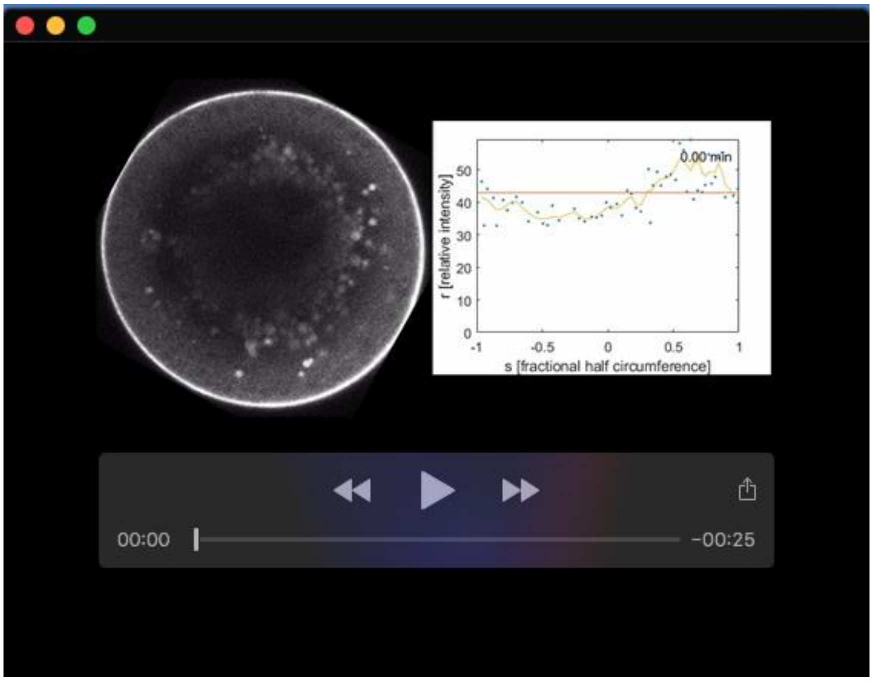

Movie 4. Time course and quantification of Med-GFP Embryos 1-5.

Biology Open • Supplementary information

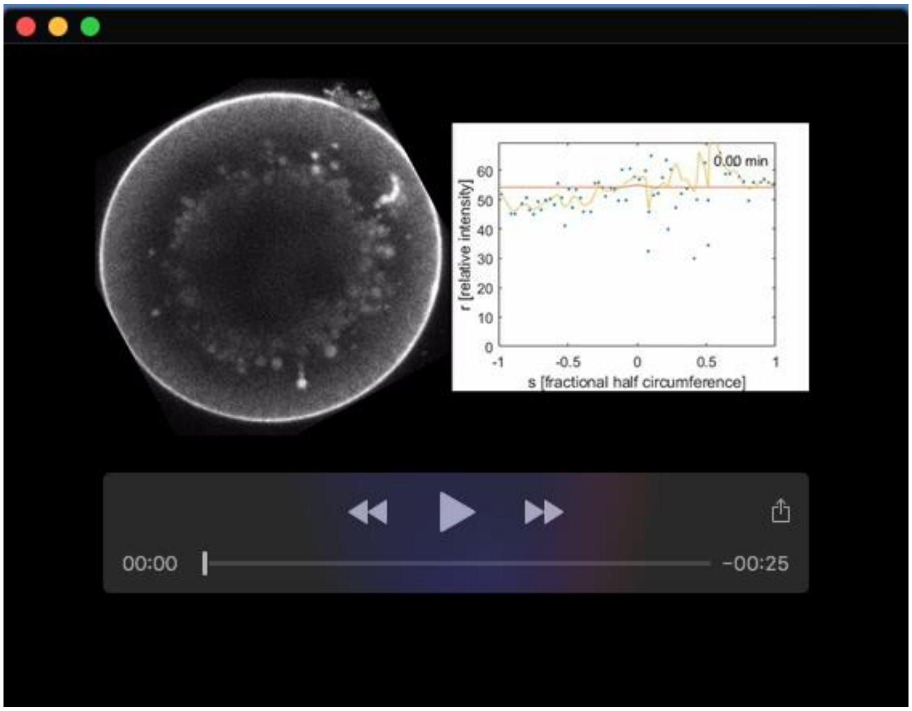

Movie 5. Time course and quantification of Med-GFP Embryos 1-5.

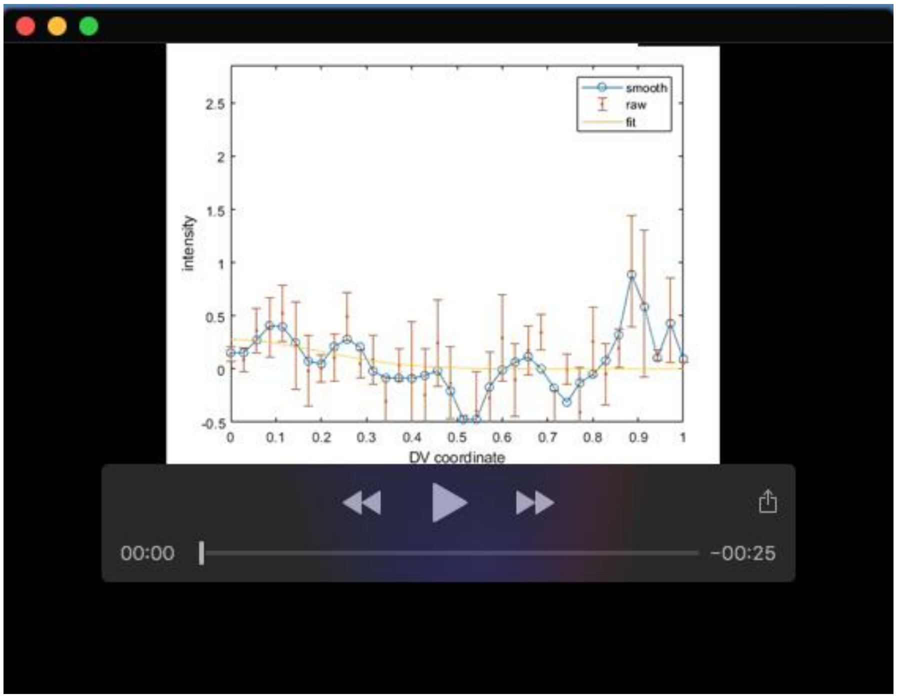

Movie 6. Graph of dynamics of Med-GFP averaged between Embryos 1-5.

Biology Open • Supplementary information

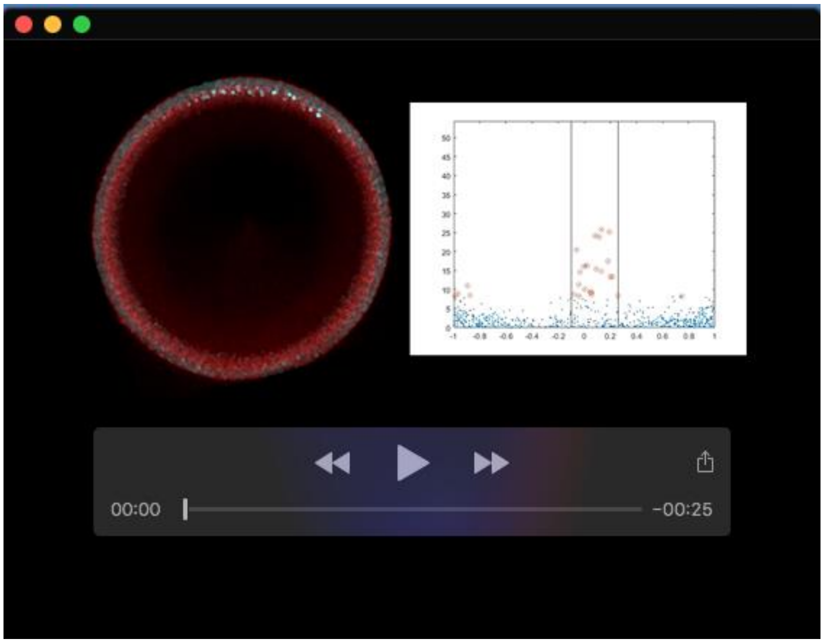

Movie 7. Time course and quantification of pnr-GFP Embryos 1-8. The time resolution of each movie is between 2 min and 3.3 min per frame.

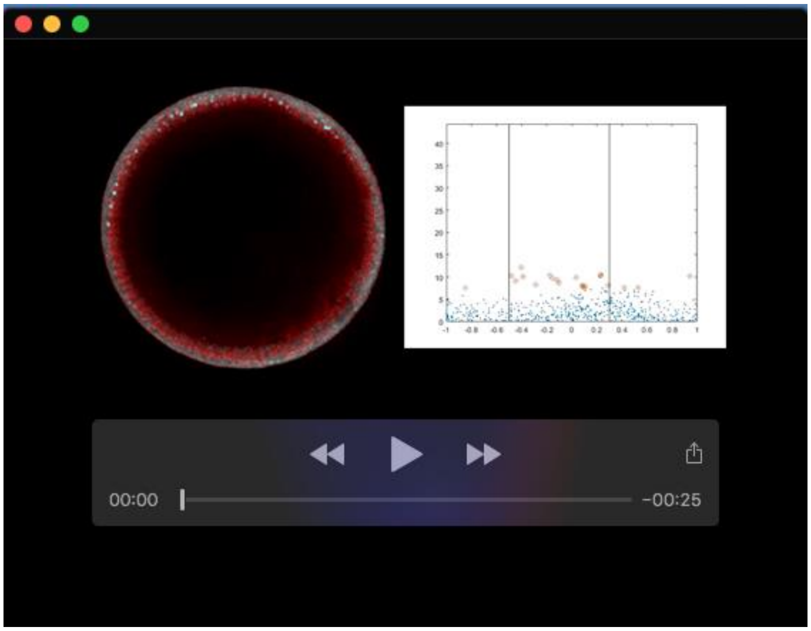

Movie 8. Time course and quantification of pnr-GFP Embryos 1-8. The time resolution of each movie is between 2 min and 3.3 min per frame.

Biology Open • Supplementary information

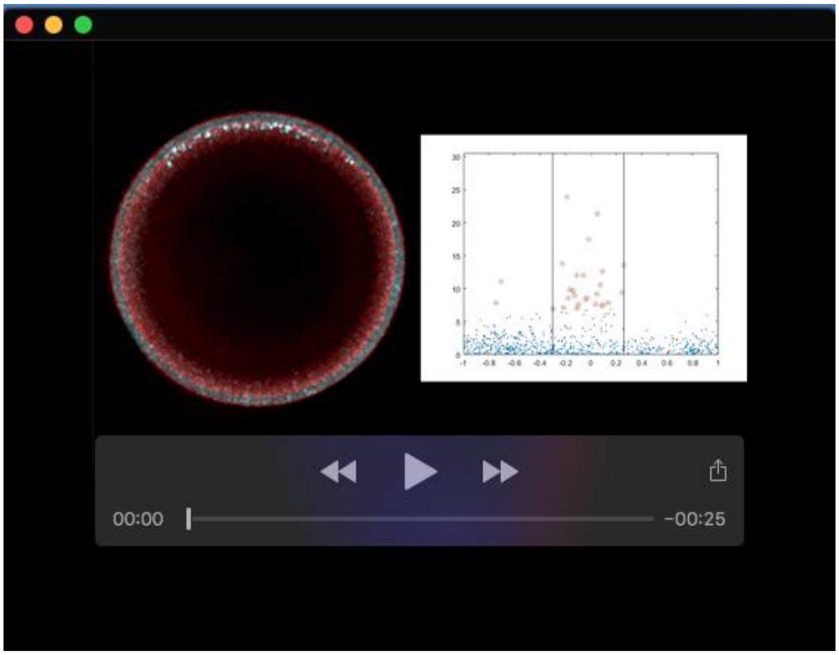

Movie 9. Time course and quantification of pnr-GFP Embryos 1-8. The time resolution of each movie is between 2 min and 3.3 min per frame.

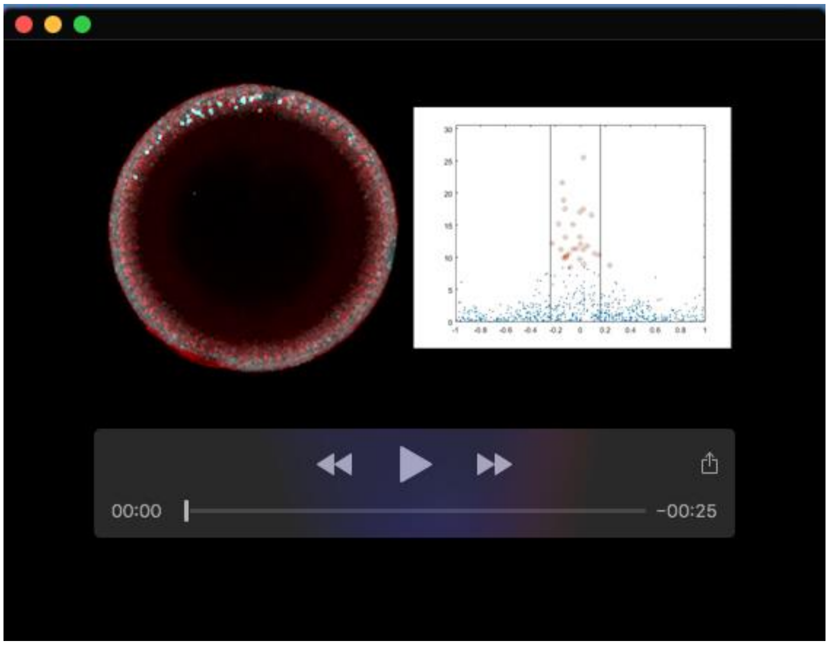

Movie 10. Time course and quantification of pnr-GFP Embryos 1-8. The time resolution of each movie is between 2 min and 3.3 min per frame.

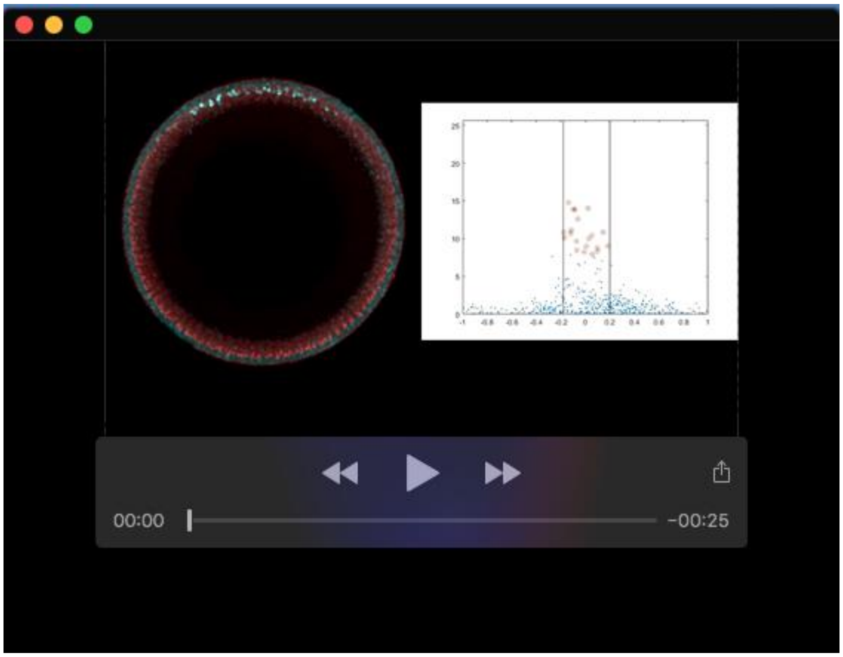

Movie 11. Time course and quantification of pnr-GFP Embryos 1-8. The time resolution of each movie is between 2 min and 3.3 min per frame.

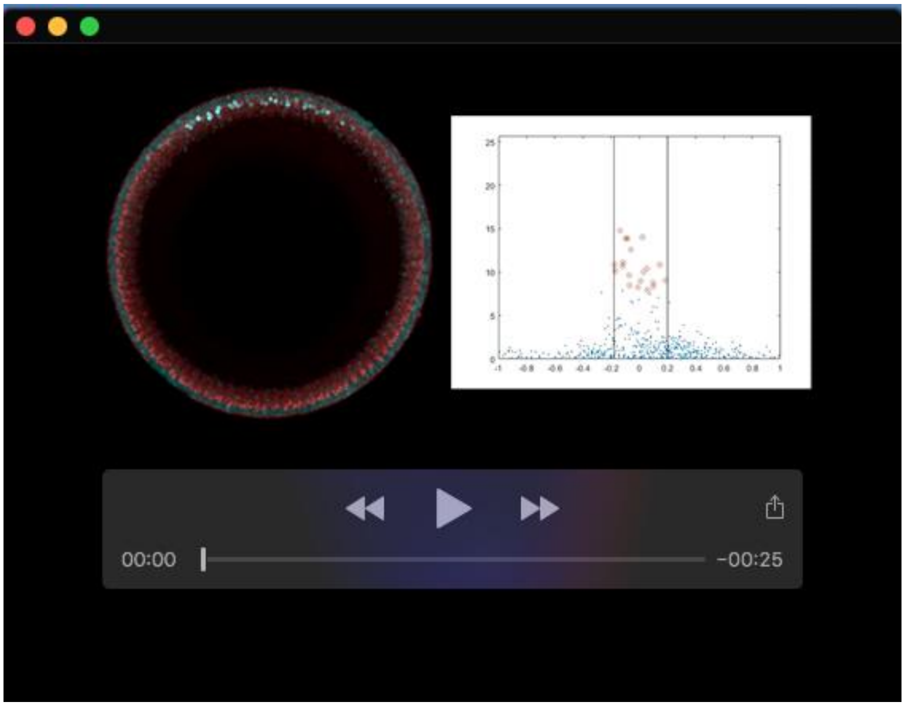

Movie 12. Time course and quantification of pnr-GFP Embryos 1-8. The time resolution of each movie is between 2 min and 3.3 min per frame.

Biology Open • Supplementary information

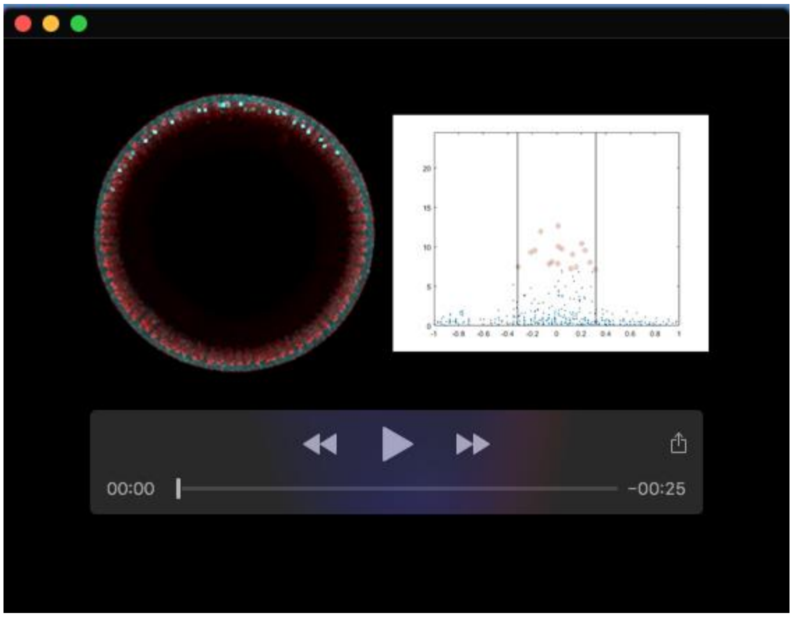

Movie 13. Time course and quantification of pnr-GFP Embryos 1-8. The time resolution of each movie is between 2 min and 3.3 min per frame.

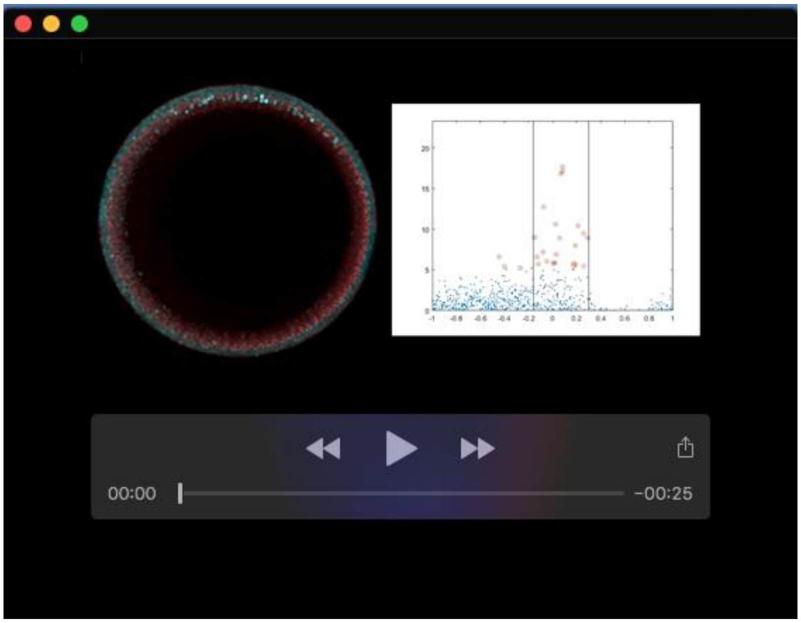

Movie 14. Time course and quantification of pnr-GFP Embryos 1-8. The time resolution of each movie is between 2 min and 3.3 min per frame.

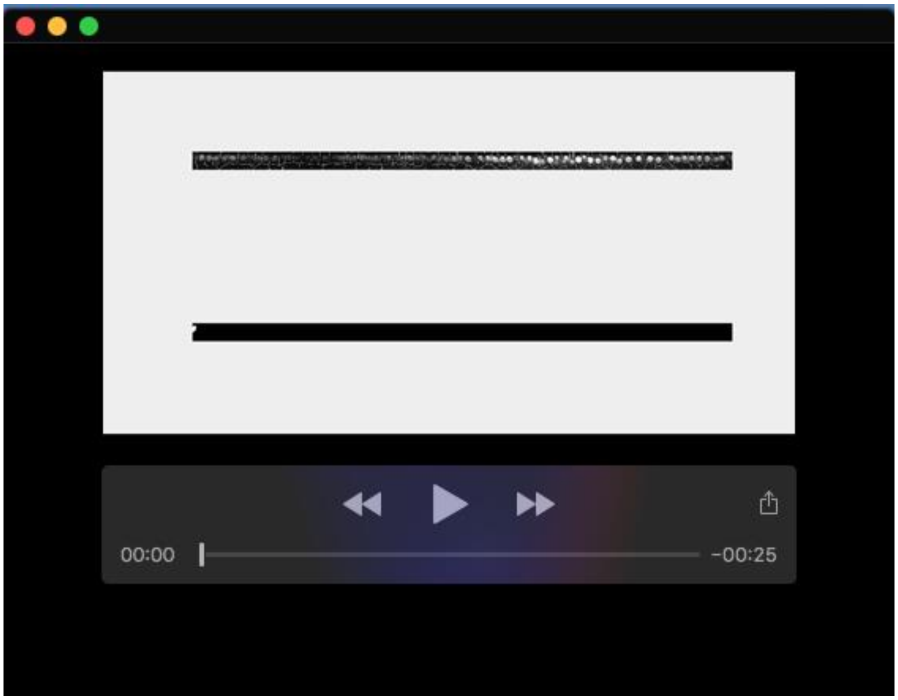

Movie 15. Image analysis of nuclear segmentation.

Biology Open • Supplementary information
